# Prebiotic, probiotic, and chlorophyllin interventions differentially attenuate melphalan-induced intestinal injury and modulate gut metabolite profiles

**DOI:** 10.3389/fmicb.2026.2003316

**Published:** 2026-10-29

**Authors:** Archana Kumari Redhu, Saithang Puia, Nitesh Chavan, Rozma Ali, Abhishek Das, Kshitij Kodolikar, Gourab Das, Sayali Kadam, Kavita Pal, Vikram Gota

**Affiliations:** 1 Department of Clinical Pharmacology, ACTREC, Tata Memorial Centre, Mumbai, India; 2 Bioinformatics and Computational Biology, Cancer Research Institute, ACTREC, Tata Memorial Centre, Mumbai, India; 3 Department of Digital & Computational Oncology, Advanced Centre for Treatment, Research and Education in Cancer (ACTREC), Tata Memorial Centre, Mumbai, India; 4 Homi Bhabha National Institute, Mumbai, India

**Keywords:** chemotherapy-induced intestinal toxicity, chlorophyllin, fructo-oligosaccharides, melphalan, probiotics, short-chain fatty acids

## Abstract

**Introduction:**

Melphalan treatment can cause substantial gastrointestinal toxicity, yet the relationship between chemotherapy-induced epithelial injury, intestinal barrier dysfunction, and alterations in the gut metabolic environment remains incompletely understood. Here, we investigated whether nutritionally and mechanistically distinct interventions could attenuate melphalan-induced intestinal injury and associated metabolic perturbations.

**Methods:**

C57BL/6 mice were allocated to vehicle control, melphalan alone, melphalan plus fructo-oligosaccharides (FOS), melphalan plus a commercial probiotic preparation containing *Lacticaseibacillus paracasei* Shirota, or melphalan plus chlorophyllin (*n* =  per group). Interventions were administered orally for 3 days before and 3 days after a single oral dose of melphalan (10 mg/kg). Intestinal toxicity was characterized through physiological outcomes, circulating markers of epithelial injury and barrier dysfunction, intestinal protein expression, and targeted LC–MS/MS profiling of short-chain fatty acids, bile acids, and indole-derived metabolites.

**Results:**

Melphalan induced body-weight loss and shortening of the small intestine, accompanied by reduced plasma citrulline and increased circulating lipopolysaccharide and occludin, consistent with enterocyte loss and impaired intestinal barrier integrity. These alterations coincided with broad reductions across multiple metabolite classes, indicating substantial disruption of the intestinal metabolic environment. FOS, the commercial probiotic preparation, and chlorophyllin attenuated body-weight loss and restored small-intestinal length and plasma-marker levels toward vehicle-control values. FOS and the probiotic preparation significantly increased selected SCFAs and bile acids, whereas the probiotic preparation and chlorophyllin significantly increased selected tryptophan-derived metabolites. Chlorophyllin also significantly increased taurocholate relative to melphalan alone.

**Discussion:**

These findings support further investigation of gut-directed nutritional interventions as strategies to mitigate chemotherapy-associated intestinal injury, with the exploratory metabolite findings requiring confirmation in larger studies.

## Graphical abstract

**Figure:**
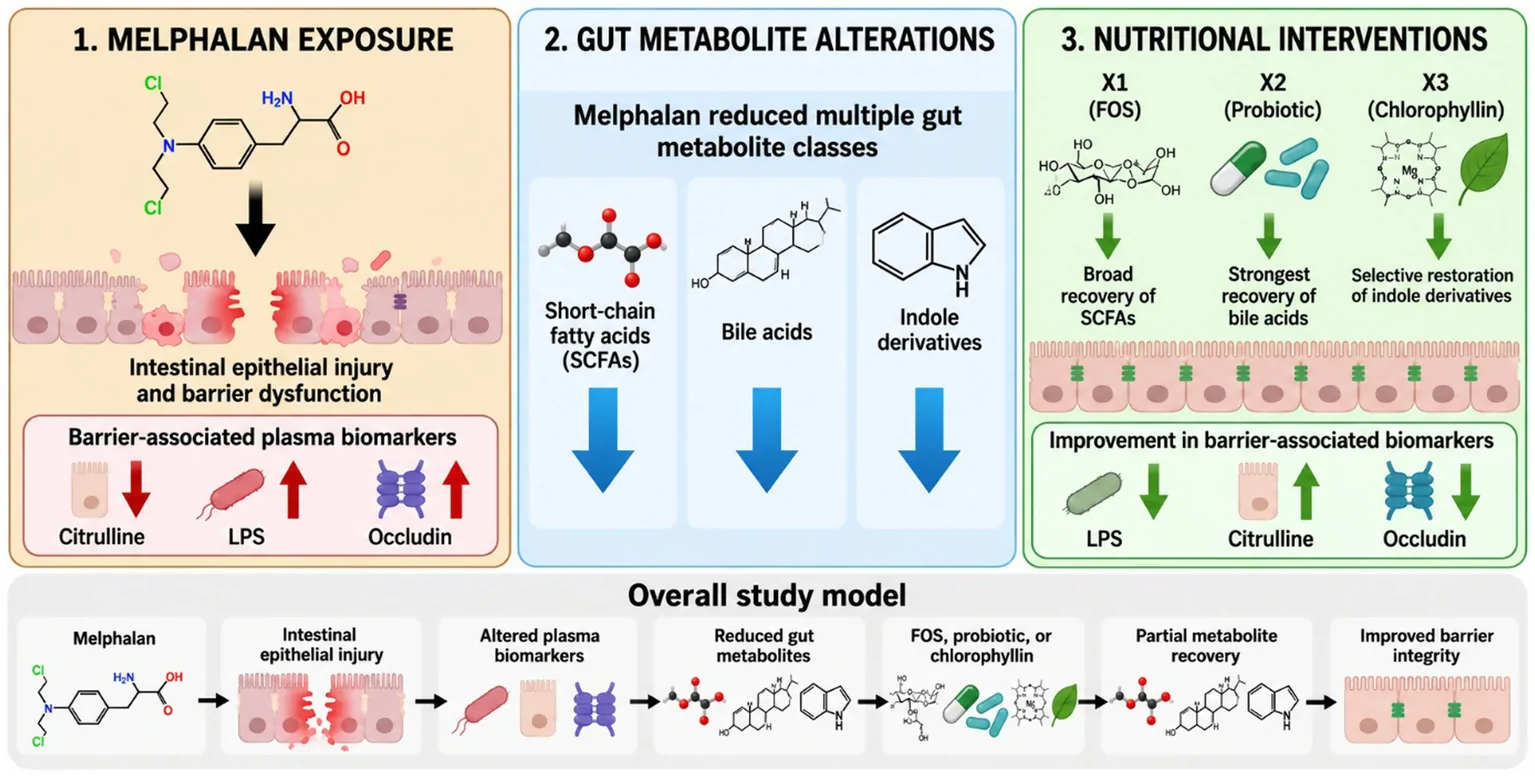

## Introduction

High-dose melphalan is an established conditioning regimen for autologous hematopoietic stem cell transplantation in patients with multiple myeloma and selected plasma cell disorders, including AL amyloidosis (Gyurkocza and Sandmaier, 2014; Mikhael et al., 2013; Palumbo et al., 2015; Palumbo et al., 2004). Despite its therapeutic efficacy, high-dose melphalan (HDM; 100–200 mg/m^2^) frequently causes gastrointestinal toxicity, with oral and intestinal mucositis reported in a substantial proportion of treated patients (van der Velden et al., 2009; Wardill et al., 2021; Akbarali et al., 2022). Damage to the intestinal epithelium can impair nutrient absorption, compromise barrier integrity, and facilitate the systemic translocation of luminal products, thereby contributing to diarrhea, inflammation, infection risk, and treatment-related morbidity.

Plasma citrulline, which reflects functional enterocyte mass, is widely used as a biomarker of intestinal epithelial injury following intensive chemotherapy (Wardill et al., 2021; Akbarali et al., 2022; Crenn et al., 2000). Increased circulating lipopolysaccharide (LPS) can indicate impaired barrier integrity and translocation of luminal bacterial products, whereas circulating occludin may provide an exploratory indicator of epithelial-junction disruption (Camilleri, 2021; Ellis et al., 2021; Conway et al., 2025). However, these barrier-associated changes have rarely been evaluated alongside alterations in intestinal metabolite profiles during melphalan-induced toxicity.

Short-chain fatty acids (SCFAs), bile acids, and indole derivatives are biologically active intestinal metabolites involved in epithelial energy metabolism, immune regulation, and maintenance of mucosal-barrier integrity (Koh et al., 2016; Hegyi et al., 2018; Venkatesh et al., 2014). Chemotherapy-associated changes in these metabolite classes may therefore accompany, or contribute to, impaired epithelial recovery and altered intestinal homeostasis (Wardill et al., 2021; Fang et al., 2016; Tian et al., 2020). Importantly, metabolite profiling provides functional information about the intestinal biochemical environment without requiring that the observed changes be attributed to alterations in microbial composition. Nevertheless, the relationship between melphalan-induced epithelial injury, barrier-associated circulating biomarkers, and gut metabolite profiles remains incompletely characterized.

Interventions that modify the intestinal environment may attenuate chemotherapy-associated gastrointestinal toxicity through complementary mechanisms. FOS and a commercial probiotic preparation containing *Lacticaseibacillus paracasei* Shirota were selected to represent prebiotic and probiotic strategies, respectively (Carvalho et al., 2021; Wongkrasant et al., 2020; Gasaly et al., 2021; Gibson et al., 2017). FOS is a fermentable prebiotic substrate that can support the production of SCFAs, including acetate, propionate, and butyrate, which participate in epithelial energy metabolism and barrier regulation (Carvalho et al., 2021; Wongkrasant et al., 2020; Gasaly et al., 2021). Probiotic preparations containing viable microorganisms have also been investigated as supportive interventions for chemotherapy-induced intestinal mucositis (Camilleri, 2021; Gasaly et al., 2021). The Shirota strain was specifically selected because it is a well-characterized probiotic with reported immunomodulatory and epithelial barrier-supporting properties (Gibson et al., 2017). Chlorophyllin is not an established prebiotic or postbiotic and was therefore included as a mechanistically distinct comparator based on its reported antioxidant, anti-inflammatory, and intestinal protective properties (Zhang et al., 2022; Kamat et al., 2000).

In the present study, we established a non-lethal mouse model of melphalan-induced intestinal toxicity and compared the potential protective effects of FOS, a commercial probiotic preparation containing *L. paracasei* Shirota, and chlorophyllin. Intestinal injury was evaluated using body weight, small-intestinal length, circulating barrier-associated biomarkers, and intestinal expression of inflammatory, junctional, and oxidative-stress proteins. Targeted metabolite profiling was performed to determine whether melphalan and the three interventions produced distinct alterations in SCFAs, bile acids, and indole derivatives. We hypothesized that these interventions would differentially attenuate melphalan-induced barrier dysfunction and partially restore the associated intestinal metabolite alterations.

## Materials and methods

### Animal ethics and experimental design

All animal experiments were approved by the Institutional Animal Ethics Committee of ACTREC, Tata Memorial Centre (Project No. 49/2022) and were conducted in accordance with the Committee for the Purpose of Control and Supervision of Experiments on Animals, Government of India. C57BL/6 mice of both sexes (6–8 weeks old; 22 ± 2 g) were acclimatized for 7 days and maintained under specific pathogen-free conditions at 22 ± 2 °C, 50–60% relative humidity, and a 12-h light–dark cycle, with standard laboratory chow and water provided ad libitum. The individual mouse was the experimental unit. The study evaluated melphalan-induced intestinal toxicity and the effects of FOS, a probiotic preparation, and chlorophyllin on physiological injury, plasma barrier-associated biomarkers, intestinal protein expression, and targeted cecal metabolites.

### Melphalan dose-optimization study

A two-stage melphalan dose-optimization study was conducted using male and female C57BL/6 mice. In the preliminary experiment, mice received oral melphalan at 10, 20, 30, 40, or 60 mg/kg. A separate validation experiment was subsequently conducted using 10 and 20 mg/kg melphalan to confirm the selection of a non-lethal dose for the main intervention study. Each dose group comprised 10 mice, including five males and five females. Animals were monitored daily for survival, body-weight loss, fecal consistency, food and water intake, general activity, and rectal temperature. Plasma citrulline, total white blood cell counts, differential leukocyte counts, and histopathological changes in the gastrointestinal tract and major vital organs were evaluated at the experimental endpoint. Dose selection was based primarily on survival and the presence of measurable treatment-related toxicity.

For clinical context, high-dose melphalan conditioning is commonly administered at 140–200 mg/m^2^ (Gyurkocza and Sandmaier, 2014; Palumbo et al., 2004). For a 70-kg adult with an estimated body-surface area of 1.82 m^2^, this corresponds numerically to approximately 3.6–5.2 mg/kg. This comparison is provided solely for clinical context and should not be interpreted as a direct cross-species dose conversion, because mg/kg scaling does not account for interspecies differences in pharmacokinetics, metabolism, or systemic drug exposure.

### Main melphalan intervention study and treatment groups

Following dose optimization, 30 mice were assigned unique identifiers, and their sex and baseline body weight were recorded. Mice were randomly allocated to five experimental groups using a random allocation sequence, which was retained in the study records. Each group initially comprised six mice, including three males and three females: vehicle control (NC), melphalan alone at 10 mg/kg (PC), melphalan plus FOS at 100 mg/kg (X1), melphalan plus probiotic preparation at 1 × 10^6^ CFU/mouse (X2), and melphalan plus chlorophyllin at 180 mg/kg (X3). One mouse in the melphalan + probiotic preparation group died during sample collection due to terminal bleeding. Body-weight analyses included six mice per group. Small-intestinal length and plasma-biomarker analyses included five mice in the probiotic group and six mice in each other group. The probiotic preparation used was Yakult® Original, a commercial fermented-milk product containing *Lacticaseibacillus paracasei* Shirota (formerly classified as *Lactobacillus casei* Shirota). The product was manufactured by Yakult Danone India Pvt. Ltd. and obtained from an authorized distributor. As a commercially available food product, it did not have a catalog number. The batch number and expiry date were recorded from the product used. The product was stored under refrigeration according to the manufacturer’s instructions and prepared immediately before administration in its original fermented-milk formulation. The administered volume was adjusted to provide 1×10^6^ CFU per mouse based on the viable-cell concentration declared by the manufacturer. FOS, the probiotic preparation, and chlorophyllin were administered orally once daily for 3 days before melphalan administration and continued for 3 days after melphalan treatment. Melphalan was administered orally as a single dose on Day 0. Animals were monitored daily for survival, body weight, food and water intake, general activity, and fecal consistency. The primary endpoints were changes in body weight, fecal consistency, food and water intake, and terminal plasma citrulline concentrations. Additional endpoints included small-intestinal length, circulating barrier-associated biomarkers, intestinal expression of inflammatory, junctional, and oxidative-stress proteins, and targeted gut metabolite profiles. The experimental design and treatment schedule are presented in Figure 1.

**Figure 1:**
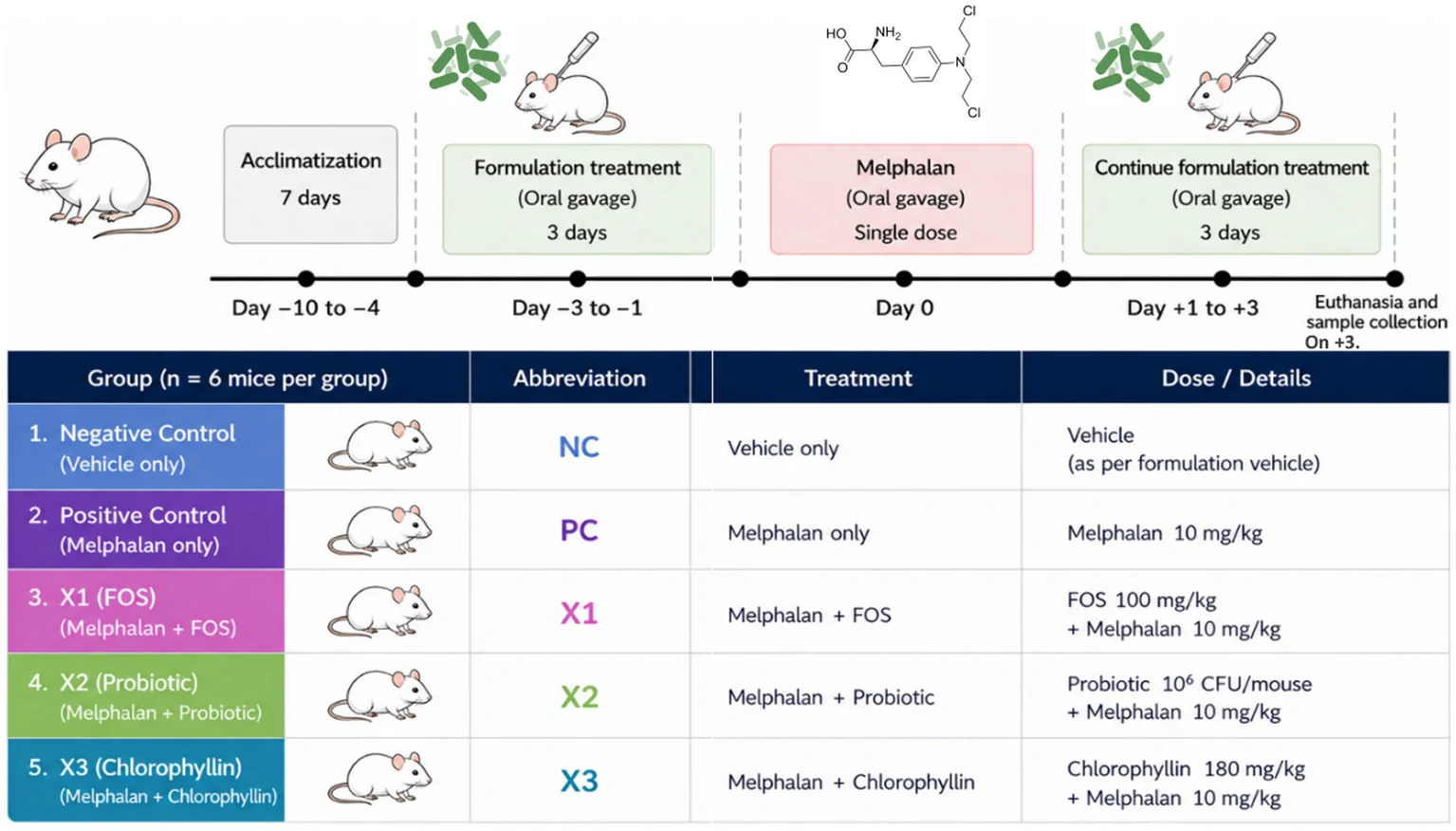
Experimental design and animal allocation. Six mice were assigned to each of five groups: vehicle control, melphalan only, melphalan + FOS, melphalan + probiotic preparation, and melphalan + chlorophyllin. Interventions were administered for 3 days before and 3 days after a single oral dose of melphalan (10 mg/kg) on Day 0.

### Sample collection and processing

On Day +3 following melphalan administration, after the final intervention dose, mice were anesthetized using isoflurane, and blood was collected from the retro-orbital sinus into EDTA-coated tubes. Animals were subsequently euthanized by cervical dislocation while maintained under deep anesthesia, and death was confirmed before tissue collection. The small intestine, colon, cecal contents, liver, lungs, heart, and brain were harvested immediately. Small-intestinal length was measured before the tissue was divided for subsequent analyses.

Blood samples were centrifuged at 2500 g for 10 min at 4 degrees to separate plasma, which was aliquoted and stored at −80 °C until analysis. Intestinal tissue intended for histopathological evaluation was fixed in 10% neutral-buffered formalin, whereas tissue intended for protein-expression analysis was snap-frozen in liquid nitrogen and stored at −80 °C. Cecal contents were collected into sterile tubes, immediately snap-frozen, and stored at −80 °C until targeted LC–MS/MS quantification of SCFAs, bile acids, and indole derivatives. Samples were maintained frozen and repeated freeze–thaw cycles were avoided. Laboratory sample assessments were performed blinded to treatment allocation using numerically coded samples, with treatment identities withheld from the assessors until assessments were completed.

### Histopathological assessment

Small-intestinal tissue sections from the main intervention experiment were assessed histologically. Samples were fixed in 10% neutral-buffered formalin, and stained with hematoxylin and eosin (H&E). Sections were evaluated by Dr. Poonam Gera (*Asst Professor and Pathologist in charge Biorepository at TMC ACTREC*) who was blinded to treatment allocation. Recorded features included epithelial lesions/villous blunting, crypt epithelial cell apoptosis, crypt loss, lamina propria inflammation, mucosal ulceration, and villous shortening. Injury scores were defined as 0, no abnormality detected; 1, mild abnormality; and 2, moderate abnormality. Crypt regeneration was recorded separately as the presence of mitotic activity and was not interpreted as an injury-severity score. Autolysis was documented, and features that could not be reliably assessed were recorded as not assessable rather than assigned a score of zero. Individual-animal findings are presented in Supplementary Data Sheet S4.

### Plasma biomarker analysis

Plasma concentrations of citrulline, occludin, and lipopolysaccharide (LPS) were measured using commercially available mouse-specific ELISA kits (GenLISA™, Krishgen Biosystems, Mumbai); according to the manufacturer’s instructions. Plasma samples from six mice per group, except the melphalan + probiotic preparation group (*n* = 5), were analyzed in technical triplicate at the recommended dilution. Absorbance was measured at 450 nm using a microplate reader. Analyte concentrations were calculated by interpolation from the standard curve generated independently for each assay and corrected for the corresponding dilution factor. Plasma citrulline was evaluated as a surrogate marker of functional enterocyte mass. Circulating LPS was used as an indicator of intestinal-barrier impairment and systemic exposure to luminal bacterial products, whereas plasma occludin was evaluated as an exploratory indicator of epithelial tight-junction disruption.

### Protein estimation and Western blotting

Protein lysates were prepared from small-intestinal tissues (*n* = 3 biological samples per group) using RIPA buffer containing protease and phosphatase inhibitors. Protein concentrations were determined using the BCA assay. Equal amounts of protein were separated by SDS-PAGE and transferred onto PVDF membranes. After blocking with 5% non-fat dry milk, membranes were incubated overnight at 4 °C with primary antibodies against TLR2 (TL2.1; sc-21759), claudin-1 (A-9; sc-166338), SOD1 (G-11; sc-17767), and phosphorylated NF-κB p65 (Ser311; A-8; sc-166748), all from Santa Cruz Biotechnology, at a dilution of 1:1,000. Membranes were subsequently incubated with the appropriate HRP-conjugated secondary antibodies: anti-mouse IgG (Cell Signalling Technology; #7076) or anti-mouse IgM (Invitrogen #62–6,820), each at a dilution of 1:5,000. Protein bands were visualized using enhanced chemiluminescence and quantified by densitometry. Band intensities were quantified by densitometry and normalized to the corresponding β-actin (C4; sc-47778; Santa Cruz Biotechnology) band intensities.

### Sample preparation for targeted metabolomic analysis

Targeted metabolite profiling was performed using an in-house-developed LC–MS/MS method based on established approaches for the extraction and quantification of metabolites in fecal samples (Kelly et al., 2022). Authentic metabolite standards were purchased from Sigma-Aldrich. Deuterated internal standards, i.e., cholic acid-d₄, indole-d₇, and acetic acid-d₄ were purchased from Toronto Research Chemicals. The derivatization reagents 3-nitrophenylhydrazine hydrochloride and EDC were purchased from Sigma-Aldrich and BLD Pharm, respectively. The method was developed and assessed for calibration linearity across the evaluated concentration ranges. Selected bile acids, indole-derived metabolites, and short-chain fatty acids (SCFAs) were quantified in cecal contents (Xia et al., 2024; Song et al., 2019; Weng et al., 2024).

Approximately 20 mg of dry-weight cecal content was extracted with 400 μL of acetonitrile:water (1:1, v/v) by vortex mixing for 2 min, sonication for 15 min, and centrifugation at 10,000 rpm for 10 min. For bile-acid and indole-derivative analysis, the resulting supernatants were spiked with the class-specific internal standards cholic acid-d4 and indole-d7, respectively. SCFAs were quantified following derivatization with 3-nitrophenylhydrazine and EDC using acetic acid-d4 as the internal standard. Derivatization-based LC–MS methods have previously been used to improve the chromatographic retention and detection sensitivity of SCFAs (Han et al., 2015; Song et al., 2019; Weng et al., 2024). The prepared extracts were subsequently subjected to targeted LC–MS/MS analysis.

### Targeted LC–MS/MS analysis of gut associated metabolites

For bile-acid and indole-derivative analysis, 10 μL of the supernatant was spiked with the corresponding class-specific isotopically labeled internal standard cholic acid-d4 for bile acids and indole-d7 for indole derivatives and diluted to a final volume of 200 μL with acetonitrile:water (1:1, v/v). For SCFA analysis, 100 μL of the supernatant was spiked with acetic acid-d4 and derivatized using 150 μL of 3-nitrophenylhydrazine (3-NPH; 200 mM) and 150 μL of EDC (120 mM). Derivatization was performed at 40 °C for 30 min and quenched with 600 μL of acetonitrile:water (1:1, v/v). Following centrifugation, the resulting supernatant was transferred to autosampler vials for analysis.

Chromatographic separation was performed using an ACQUITY UPLC H-Class Plus system fitted with an ACQUITY UPLC HSS T3 column (100 × 2.1 mm, 1.8 μm; Waters Corporation, Milford, MA, USA) maintained at 40 °C. The flow rate was 0.4 mL/min, and the injection volume was 5 μL. Mobile phase A consisted of 10 mM ammonium formate containing 0.1% formic acid, and mobile phase B consisted of acetonitrile. Metabolites were detected using a Xevo™ TQ Absolute triple-quadrupole mass spectrometer (Waters Corporation) equipped with an electrospray-ionization source operating in positive- and negative-ion multiple-reaction-monitoring modes. Data were acquired and processed using MassLynx™ software.

The analytical method was developed and assessed for calibration linearity using matrix-matched calibration standards. Calibration curves were generated from analyte-to-internal-standard peak-area ratios and fitted using weighted 1/x^2 least-squares linear regression. Class-specific isotopically labeled internal standards were added to calibration standards, quality-control samples, and study samples. All calibration curves demonstrated linear responses with (r^2 = 0.999) across the evaluated concentration ranges. Deoxycholic acid was linear over 0.5–1,600 μg/g dry weight, whereas lithocholic acid, taurocholic acid, and cholic acid were linear over 1.0–267 μg/g dry weight. Indole-derived metabolites were linear over 0.5–267 μg/g dry weight, and SCFAs were linear over 3–80,000 μg/g dry weight. Detailed chromatographic gradients, source parameters, retention times, MRM transitions, calibration equations and ranges, method-development and linearity-assessment data, and representative chromatograms, metabolites external standards, lot numbers, internal standards and derivatisation details are provided in Supplementary Data Sheet S1–S3.

### Statistical analysis

Statistical analyses were performed using GraphPad Prism version 10.0. Sample size was estimated *a priori* using G*Power with α = 0.05. Both sexes were included, and each mouse was considered an experimental unit. Data are presented as mean ± SEM or mean ± SD, as specified in the corresponding figure legends. Normality was assessed using the Shapiro–Wilk test. Percentage body-weight change from Day 0 was analyzed using two-way repeated-measures ANOVA with Geisser–Greenhouse correction, followed by Dunnett’s multiple-comparisons test. Plasma biomarkers and other endpoint measurements were analyzed separately using one-way ANOVA followed by Dunnett’s multiple-comparisons test. When parametric assumptions were not satisfied, the Kruskal–Wallis test followed by Dunn’s multiple-comparisons test was used. Prespecified comparisons were melphalan only versus vehicle control and each intervention group versus melphalan only.

For each metabolite, individual-sample log₂ fold-change values calculated relative to the mean concentration in the melphalan-only group were plotted as individual biological replicates with mean ± SD (*n* = 3 per group). These values were analyzed using one-way ANOVA followed by Dunnett’s multiple-comparisons test, with the melphalan-only group as the comparator. Multiple-comparison adjustment was applied within each metabolite; no additional adjustment across metabolites was performed. Accordingly, metabolite findings were interpreted as exploratory. Spearman’s rank correlation was used to assess the association between paired individual-animal plasma citrulline and LPS measurements.

The body-weight (*n* = 6), gross-anatomical, and plasma biomarker analyses included six mice in each group except the melphalan + probiotic preparation group, which included five mice. Cecal samples containing <50 mg of material were excluded before LC–MS/MS analysis because insufficient material was available for standardized extraction and quality-control analysis. This objective criterion was applied uniformly across groups and without knowledge of the analytical results, leaving three samples per group (*n* = 3). No additional samples were excluded. The main experiment was conducted once. All statistical tests were two-sided, and *p* < 0.05 was considered statistically significant.

## Results

### Selection of melphalan dose for the gut injury model

Melphalan dose optimization was performed in C57BL/6 mice using doses of 10, 20, 30, 40, and 60 mg/kg, with 10 mice per dose group (five males and five females). Doses of 30–60 mg/kg resulted in mortality within 2–4 days following melphalan administration. In a separate validation experiment, the 20 mg/kg dose also resulted in mortality by Day 4. In contrast, no mortality was observed in the 10 mg/kg group during the observation period. The 10 mg/kg dose produced measurable body-weight loss and reduced feed intake without mortality and was therefore selected for the subsequent intervention study as a non-lethal dose capable of inducing measurable toxicity.

### Effects of interventions on melphalan-associated physiological changes

Melphalan-treated mice showed progressive body-weight loss, whereas the intervention groups showed recovery toward baseline following an initial decline (Figure 2a). There were significant effects of time [*F*(3.332, 83.31) = 11.80, *p* < 0.0001] and time × treatment [*F*(24, 150) = 4.419, *p* < 0.0001], but no overall treatment effect [*F*(4, 25) = 1.744, *p* = 0.1718]. Dunnett-adjusted comparisons identified less weight loss with FOS on Day 3 (*p* = 0.0262) and greater weight loss with chlorophyllin on Day 1 (*p* = 0.0288) relative to melphalan alone. Vehicle-control mice also differed from melphalan-only mice on Days 2 and 3 (adjusted *p* = 0.0428 and 0.0086, respectively). The remaining evaluable comparisons against melphalan alone were not significant (adjusted *p* > 0.05). Small-intestinal length was lower in the melphalan-only group than in vehicle controls and higher in the FOS and probiotic groups than in the melphalan-only group; these comparisons were significant, whereas the chlorophyllin comparison was not (Figure 2b). Food and water intake showed differing trajectories across treatment groups (Supplementary Figures 1A,B). Following melphalan administration on Day 0, food intake declined in the melphalan-only group, remained relatively stable in the FOS group, and increased in the chlorophyllin group. The probiotic group showed lower food intake throughout the observation period, including before melphalan administration. Water intake declined after Day 0 in the melphalan-only group, whereas the intervention groups showed variable trajectories, including increases between Days +1 and +3 in the FOS and probiotic groups. These observations were assessed descriptively. Differences in intake were already apparent before melphalan administration, limiting attribution of the subsequent trajectories to treatment effects. No diarrhea was observed during the main intervention experiment. Semi-solid feces were noted in the melphalan + chlorophyllin group. To assess small-intestinal histopathological changes in the main intervention experiment, H&E-stained tissue sections were examined. Melphalan-only animals showed lamina propria inflammation, mucosal ulceration, and occasional villous shortening compared with largely normal vehicle-control findings. Histopathological abnormalities, including crypt loss, were also recorded in the intervention groups, without consistent normalization at Day +3 (Supplementary Data Sheet S4).

**Figure 2:**
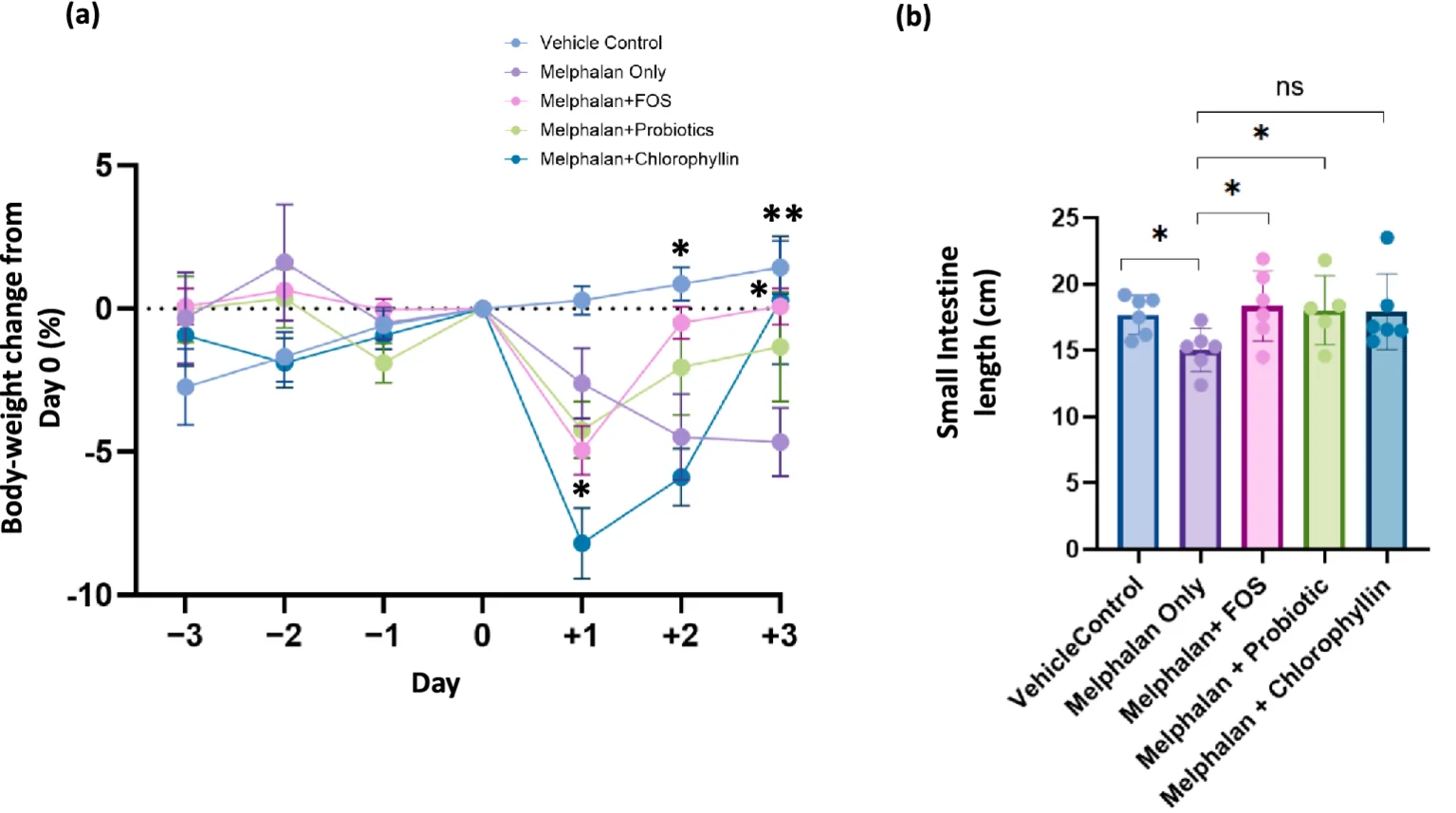
Body-weight trajectories and small-intestinal length. **(a)** Percentage body-weight change from Day 0 (melphalan administration). **(b)** Endpoint small-intestinal length, with individual values overlaid. Data are mean ± SEM; *n* = 6/group, except *n* = 5 for probiotic in B because one mouse died during sample collection after all weight measurements. **(a)** Two-way repeated-measures ANOVA with Geisser–Greenhouse correction for time; **(b)** one-way ANOVA. Both were followed by Dunnett’s comparisons versus melphalan alone, within each day for A. *Adjusted *p* < 0.05; **adjusted *p* < 0.01; ns, not significant.

### Test formulations attenuate melphalan-induced intestinal barrier dysfunction

Compared with the vehicle control, melphalan reduced plasma citrulline and increased circulating LPS and occludin (Figures 3a–c). These changes were consistent with loss of functional enterocyte mass, increased systemic exposure to luminal bacterial products, and disruption of epithelial tight junctions. Treatment with FOS, the probiotic preparation, or chlorophyllin generally increased citrulline and reduced LPS and occludin relative to melphalan alone, although the magnitude and statistical significance of these effects varied across interventions and biomarkers. The probiotic group exhibited the lowest mean LPS concentration among the intervention groups, although all three interventions showed improvement in markers associated with epithelial injury and barrier dysfunction. Collectively, the plasma biomarker profiles showed that all three interventions attenuated melphalan-associated epithelial injury and barrier dysfunction.

**Figure 3:**
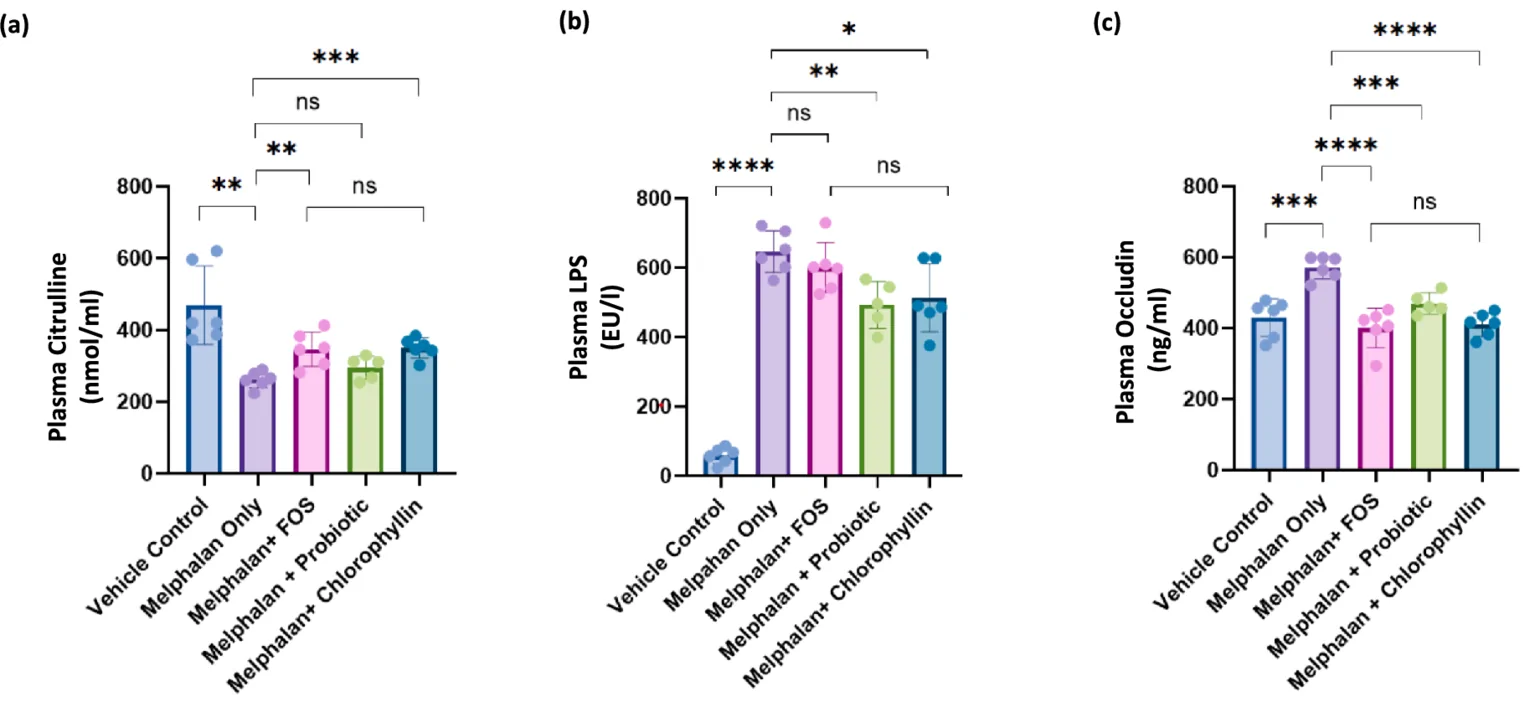
Plasma markers of intestinal injury and barrier disruption. Individual values for **(a)** citrulline, **(b)** lipopolysaccharide, and **(c)** occludin in the vehicle-control, melphalan-only, melphalan + FOS, melphalan + probiotic preparation, and melphalan + chlorophyllin groups. Data are presented as mean ± SEM, with *n* = 5 mice in the melphalan + probiotic preparation group and *n* = 6 mice in each other group. Each dot represents one individual mouse. Statistical significance was assessed separately for each biomarker using one-way ANOVA followed by Dunnett’s multiple-comparisons test. Prespecified comparisons were melphalan only versus vehicle control and each intervention group versus melphalan only. *p < 0.05, **p < 0.01, ***p < 0.001, and ****p < 0.0001; ns, not significant.

### Barrier-associated protein expression following melphalan and test formulations

To further characterize molecular alterations associated with intestinal barrier dysfunction, protein expression was assessed in small-intestinal tissue by Western blotting (Supplementary Figure 2; *n* = 3 biological samples per group). The blots suggested increased TLR2 and reduced claudin-1 expression following melphalan treatment compared with vehicle control, with partial reversal of these patterns following chlorophyllin treatment. SOD1 expression also appeared higher following melphalan treatment and comparatively lower with chlorophyllin, whereas phosphorylated NF-κB showed modest variation across groups. These descriptive findings suggest chlorophyllin-associated changes in proteins involved in barrier integrity, inflammatory signaling, and antioxidant defence.

### Melphalan-induced intestinal toxicity was associated with distinct gut metabolic alterations

Targeted LC–MS/MS profiling was performed to determine whether melphalan exposure and the three interventions were associated with alterations in selected bile acids, indole-derived tryptophan metabolites, and short-chain fatty acids (SCFAs). These metabolite classes were examined because of their established roles in epithelial energy metabolism, mucosal immune regulation, and maintenance of intestinal-barrier integrity. The developed LC–MS/MS method demonstrated linear calibration responses across the evaluated concentration ranges, with coefficients of determination of r^2^ ≥ 0.99 for all quantified analytes. Retention times, precursor/product-ion transitions, cone voltages, and collision energies are provided in Supplementary Data Sheet S1. Detailed method-development, calibration and analytical-performance data are provided in Supplementary Data Sheet S2. Class-specific isotopically labeled internal standards were used to account for analytical variability, while 3-nitrophenylhydrazine derivatization improved the chromatographic retention and detection sensitivity of SCFAs (Supplementary Data Sheet S3).

A global overview of cecal metabolite profiles is presented in Figure 4. The melphalan-only group was selected as the common reference because the primary objective was to evaluate intervention-associated changes relative to the melphalan-induced injury condition. For each metabolite, individual-sample log₂ fold changes were calculated as log₂ (individual sample concentration/mean concentration in the melphalan-only group). Applying this same reference across all five groups allows the direction and magnitude of metabolite differences to be compared consistently. Compared with vehicle control, the melphalan-only group showed lower levels across the measured bile acids, tryptophan-derived metabolites, and SCFAs. FOS and the commercial probiotic preparation showed positive shifts across several metabolites relative to melphalan alone, whereas chlorophyllin showed a more selective pattern, including higher levels of the measured tryptophan-derived metabolites and acetate. The magnitude and direction of these differences varied across interventions and metabolite classes. The heatmap provides a descriptive overview of these patterns; statistical comparisons are reported in the class-specific analyses of bile acids, tryptophan-derived metabolites, and SCFAs (Figures 5–7).

**Figure 4:**
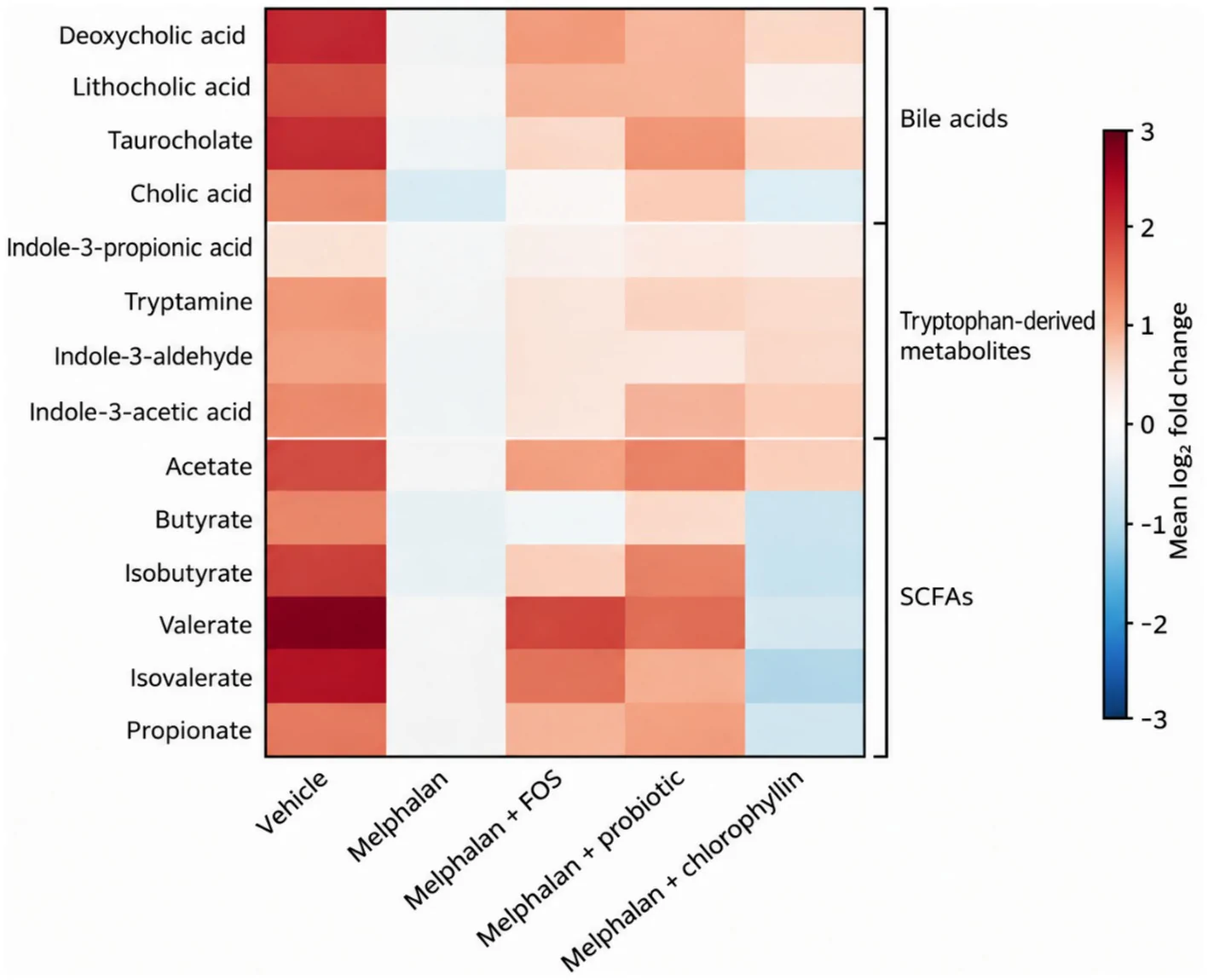
Heatmap of mean individual-sample log₂ fold changes in targeted cecal metabolites. Each cell represents the mean of three individual-sample log₂ fold changes (*n* = 3 biological samples per group). For each metabolite, values were calculated as log₂ (individual sample concentration/mean concentration in the Melphalan-only group), using the same reference across all five groups. Red indicates positive values, blue indicates negative values, and near-white indicates values close to zero. Colors represent the magnitude of log₂ fold changes, not statistical significance.

**Figure 5:**
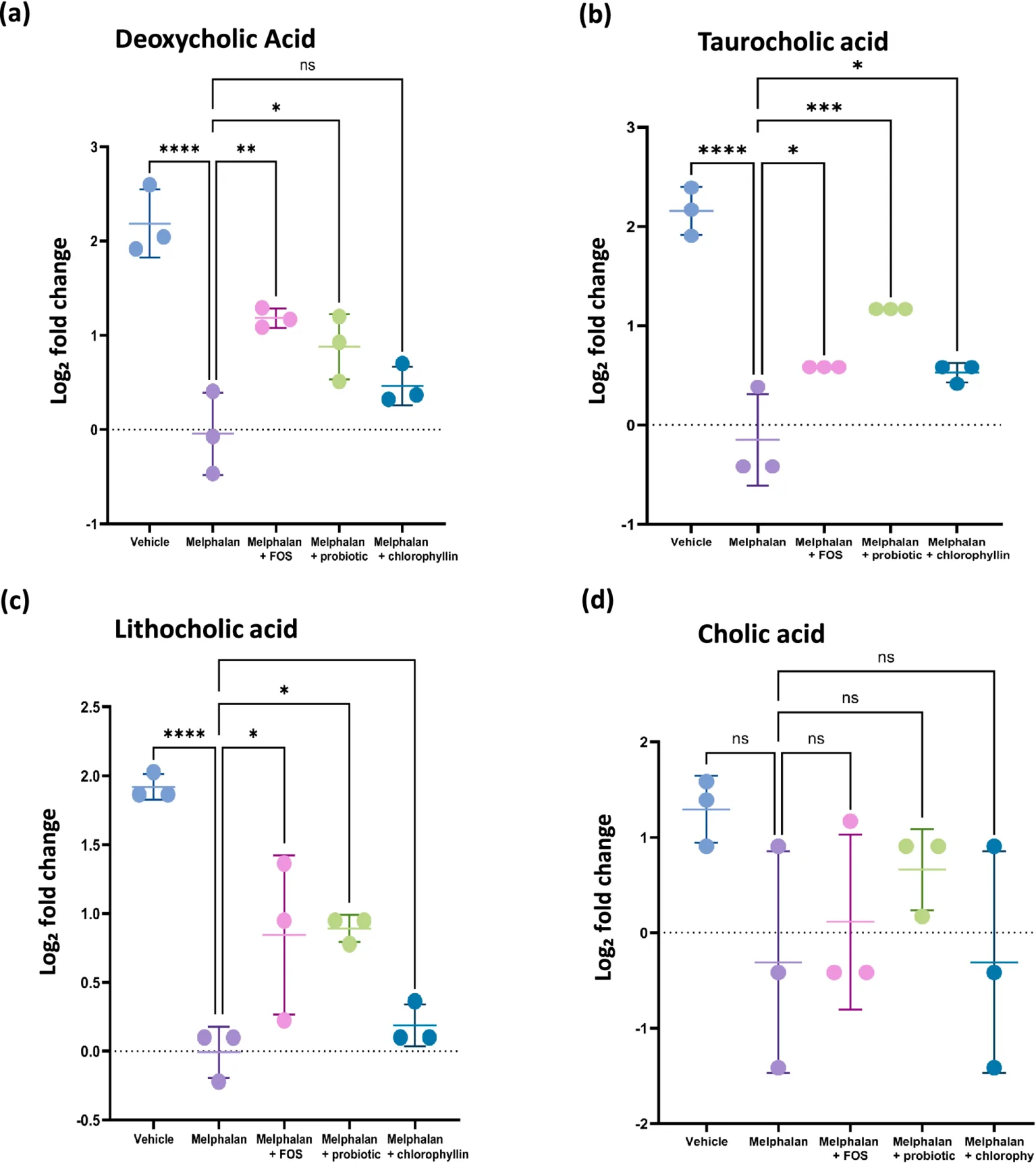
Mean log₂ fold changes in cecal bile acids following melphalan and intervention treatments: **(a)** Deoxycholic acid; **(b)** Taurocholic acid; **(c)** Lithocholic acid; and **(d)** Cholic acid. Individual biological samples are shown with mean log₂ fold change ± SD (*n* = 3 per group). For each metabolite, log₂ fold change was calculated as log₂ (individual sample concentration/mean concentration in the melphalan-only group), using the same reference across all five groups. Statistical significance was assessed using one-way ANOVA on individual-sample log₂-transformed values followed by Dunnett’s multiple-comparisons test, with the melphalan-only group as the comparator for all other groups within each metabolite. Asterisks indicate *p*-values adjusted for multiple comparisons within each metabolite: *p < 0.05, **p < 0.01, ***p < 0.001, and ****p < 0.0001; ns, not significant. FOS, fructooligosaccharides.

**Figure 6:**
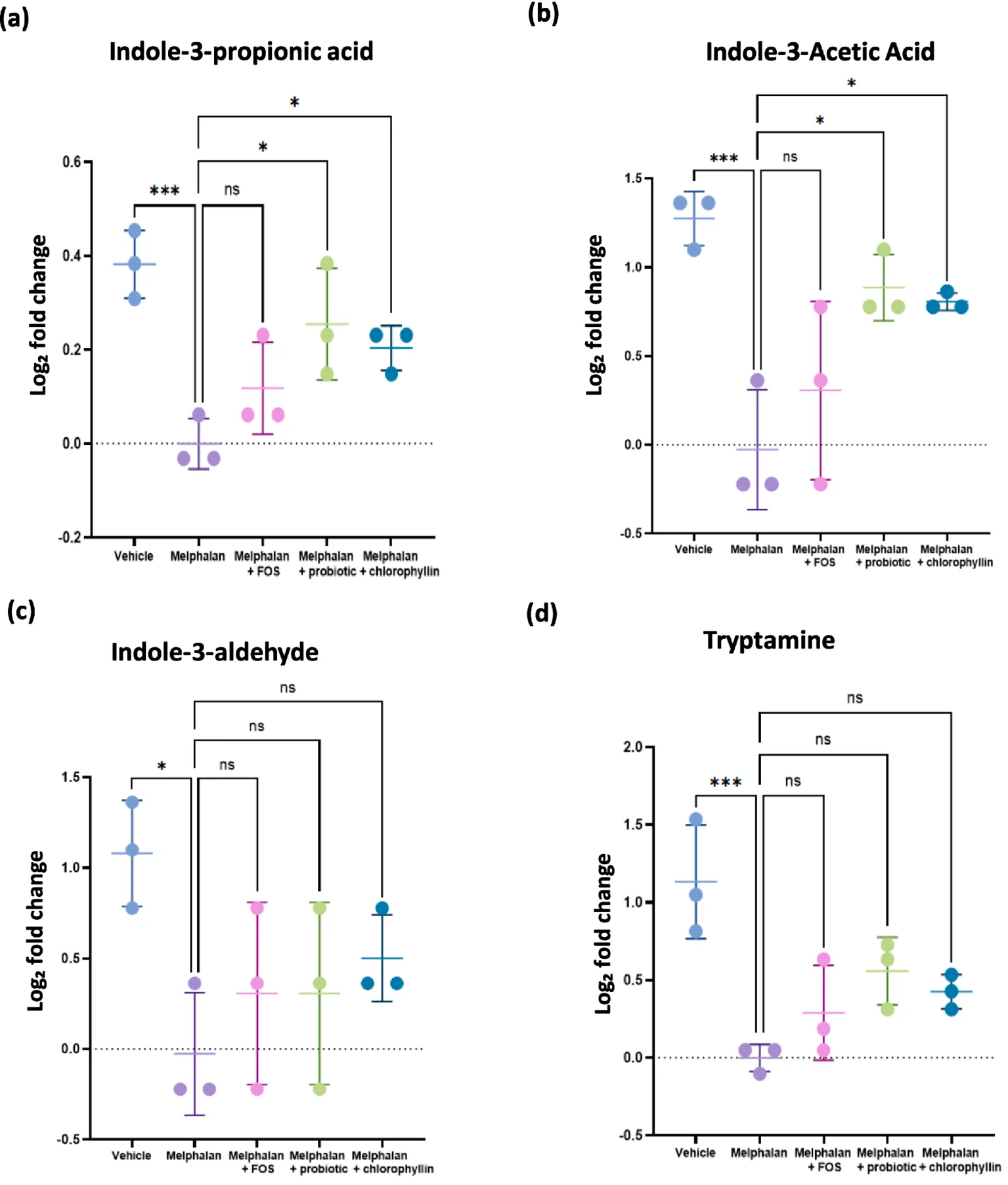
Mean log₂ fold changes in cecal tryptophan-derived metabolites following melphalan and intervention treatments. **(a)** Indole-3-propionic acid; **(b)** Indole-3-acetic acid; **(c)** Indole-3-aldehyde; and **(d)** Tryptamine. Individual biological samples are shown with mean log₂ fold change ± SD (*n* = 3 per group). For each metabolite, log₂ fold change was calculated as log₂ (individual sample concentration/mean concentration in the melphalan-only group), using the same reference across all five groups. Statistical significance was assessed using one-way ANOVA followed by Dunnett’s multiple-comparisons test, with the melphalan-only group as the comparator for all other groups within each metabolite. Asterisks indicate *p*-val*ues adjusted for multip*le comparisons within each metabolite: *p < 0.05, **p < 0.01, ***p < 0.001, and ****p < 0.0001; ns, not significant. FOS, fructooligosaccharides.

**Figure 7:**
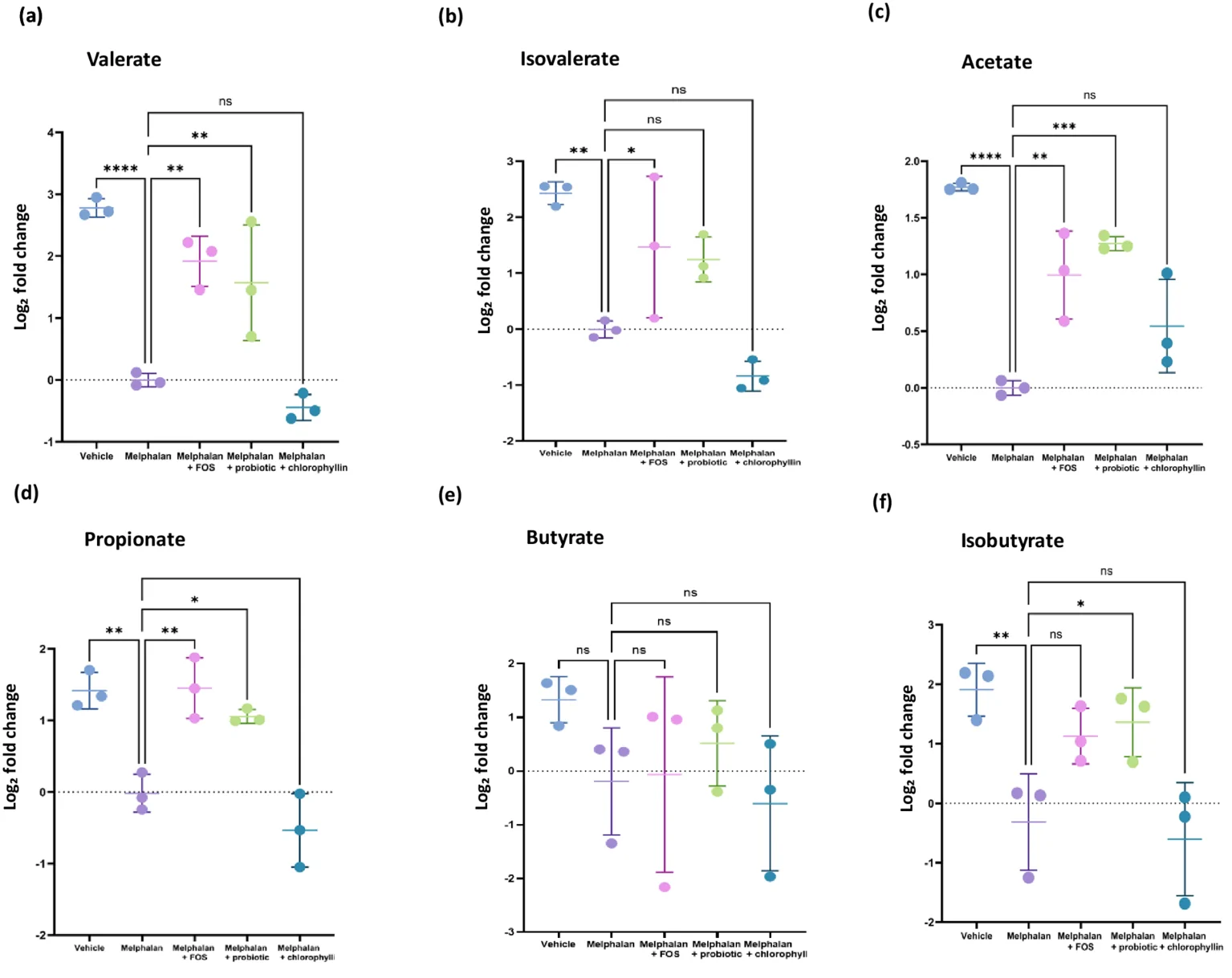
Mean log_2_ fold changes in short-chain fatty acids following melphalan and intervention treatments. **(a)** Valerate; **(b)** isovalerate; **(c)** acetate; **(d)** propionate; **(e)** butyrate; and **(f)** isobutyrate. For each metabolite, individual-sample log₂ fold changes were calculated by dividing each sample’s concentration by the arithmetic mean concentration of the melphalan-only group and applying a log₂ transformation. Data are presented as mean log₂ fold change ± SD, with individual biological samples shown (*n* = 3 per group). Statistical significance was assessed using one-way ANOVA on individual-sample log₂-transformed values followed by Dunnett’s multiple-comparisons test, with the melphalan-only group as the comparator for all other groups within each metabolite. *p < 0.05, **p < 0.01, ***p < 0.001, and ****p < 0.0001; ns, not significant.

### Interventions differentially restore melphalan-disrupted intestinal bile acid profiles

Targeted metabolite analysis showed differences in cecal bile acid profiles following melphalan administration (Figure 5). Deoxycholic acid, taurocholate, and lithocholic acid levels were significantly lower in the melphalan-only group than in the vehicle-control group (Figures 5a–c). Cholic acid levels were also lower following melphalan exposure, although this difference was not statistically significant (Figure 5d).

The interventions produced metabolite-specific responses. FOS and the commercial probiotic preparation significantly increased deoxycholic acid and lithocholic acid levels compared with melphalan alone, whereas the corresponding differences with chlorophyllin were not significant (Figures 5a,c). Taurocholate levels were significantly higher in all three intervention groups than in the melphalan-only group (Figure 5b). None of the interventions produced a significant difference in cholic acid levels compared with melphalan alone (Figure 5d). These findings indicate that significant increases occurred across more measured bile acids following FOS and probiotic treatment than following chlorophyllin.

### Interventions differentially restore melphalan-disrupted intestinal indole-derived tryptophan metabolite profiles

Melphalan administration significantly reduced cecal levels of indole-3-propionic acid, indole-3-acetic acid, indole-3-aldehyde, and tryptamine compared with the vehicle-control group (Figures 6a–d). These reductions extended across all four measured tryptophan-derived metabolites, although their magnitude varied across metabolites.

The interventions produced distinct responses. The commercial probiotic preparation and chlorophyllin significantly increased indole-3-propionic acid and indole-3-acetic acid levels compared with melphalan alone, whereas the increases following FOS were not statistically significant (Figures 6a,b). Indole-3-aldehyde and tryptamine levels were higher in the intervention groups, but none of these comparisons with melphalan alone reached statistical significance (Figures 6c,d). These findings indicate selective increases in indole-3-propionic acid and indole-3-acetic acid following probiotic and chlorophyllin treatment.

### Interventions differentially restore melphalan-disrupted intestinal short-chain fatty acid profiles

Melphalan administration altered cecal short-chain fatty acid profiles (Figure 7). Valerate, isovalerate, acetate, propionate, and iso-butyrate levels were significantly lower in the melphalan-only group than in the vehicle-control group (Figures 7a–d,f), whereas the difference in butyrate was not statistically significant (Figure 7e). The interventions produced metabolite-specific responses relative to melphalan alone. FOS and the probiotic preparation significantly increased valerate (Figure 7a). FOS also significantly increased isovalerate, whereas the probiotic preparation and chlorophyllin did not produce significant differences in this metabolite (Figure 7b). Both FOS and the probiotic preparation significantly increased acetate and propionate (Figures 7c,d). No intervention produced a significant difference in butyrate (Figure 7e), while isobutyrate was significantly increased only by the probiotic preparation (Figure 7f). Chlorophyllin did not produce statistically significant differences in any of the six metabolites.

Overall, these findings support partial recovery of selected short-chain fatty acids following FOS and probiotic treatment, with responses varying across metabolites.

### Associations between plasma biomarkers and selected intestinal metabolites

To examine the relationship between intestinal metabolic alterations and host barrier dysfunction, selected metabolites were evaluated alongside circulating barrier-associated biomarkers. Melphalan-induced reductions in acetate, butyrate, and indole-3-acetic acid occurred concurrently with decreased plasma citrulline and increased circulating LPS, consistent with intestinal epithelial injury and impaired barrier integrity. Following intervention treatment, partial recovery of these metabolites was accompanied by higher citrulline and lower LPS concentrations. Spearman’s rank-correlation analysis of paired individual-animal measurements pooled across the five treatment groups revealed a significant inverse association between plasma citrulline and LPS (rₛ = −0.5493, *p* = 0.002; Figure 8a), indicating that lower plasma citrulline concentrations were associated with higher circulating LPS concentrations. This association may partly reflect differences between treatment groups and does not establish a causal relationship. Descriptive group-level patterns of indole-3-acetic acid and butyrate also paralleled changes in plasma citrulline across the treatment groups (Figures 8b,c), although these comparisons were not used for inferential correlation analysis. Together, these findings show that changes in measured cecal metabolites occurred alongside changes in barrier-associated host biomarkers, without establishing a causal relationship between metabolic alterations and epithelial recovery.

**Figure 8:**
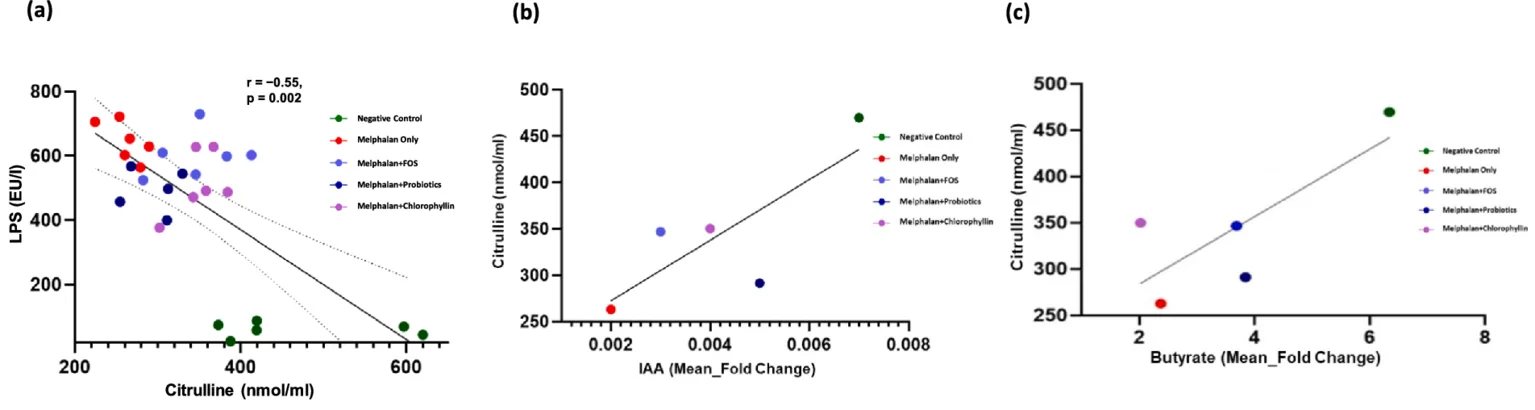
Associations between plasma markers and selected metabolites. **(a)** Spearman correlation between paired individual-animal plasma LPS and citrulline measurements (*n* = 29 mice: five in the melphalan + probiotic preparation group and six in each other group). Each point represents one mouse *r*s = −0.5493, *p* = 0.002. **(b,c)** Descriptive plots showing group-mean fold changes in indole-3-acetic acid or butyrate against group-mean plasma citrulline. **(b,c)** Contain five group-level observations, representing the five treatment groups, and were not used for inferential correlation analysis.

## Discussion

The present study demonstrates that melphalan-induced intestinal toxicity is characterized by coordinated physiological deterioration, epithelial injury, barrier dysfunction, and disruption of the intestinal metabolic environment. Melphalan reduced body weight and small-intestinal length, decreased plasma citrulline, and increased circulating LPS and occludin. These changes were accompanied by altered expression of junctional, inflammatory, and oxidative-stress-associated proteins and broad reductions in SCFAs, bile acids, and indole-derived tryptophan metabolites. Treatment with FOS, the probiotic preparation containing *L. paracasei* Shirota, or chlorophyllin partially attenuated several features of melphalan-associated toxicity, although the interventions produced distinct patterns of metabolic recovery. Collectively, these findings indicate that disruption of intestinal barrier integrity and the local metabolic environment are concurrent features of melphalan-induced mucosal injury and support further investigation of gut-directed interventions as potential strategies for mitigating chemotherapy-associated intestinal toxicity.

The reduction in plasma citrulline following melphalan administration is consistent with loss of functional enterocyte mass and mucosal-barrier injury. Citrulline is produced predominantly by small-intestinal enterocytes and is an established marker of epithelial injury following intensive chemotherapy and conditioning for hematopoietic stem-cell transplantation (Wardill et al., 2021; Crenn et al., 2000; Blijlevens et al., 2004). The concomitant increase in circulating LPS indicates greater systemic exposure to luminal bacterial products, while elevated plasma occludin is compatible with epithelial-junction disruption. These changes were accompanied by reduced intestinal claudin-1 and increased TLR2 and SOD1 expression, consistent with junctional disruption, inflammatory signaling, and redox stress. Previous melphalan models have similarly demonstrated intestinal architectural damage, inflammatory activation, reduced plasma citrulline, weight loss, and impaired gastrointestinal function (Wardill et al., 2021; Wardill et al., 2022), supporting the clinical relevance of the present non-lethal model.

Targeted LC–MS/MS revealed coordinated reductions in intestinal SCFAs, bile acids, and indole derivatives following melphalan exposure. SCFAs, particularly acetate, propionate, and butyrate, support epithelial energy metabolism, junctional integrity, mucin production, and immune regulation (Koh et al., 2016; Scott et al., 2020); their depletion therefore reflects an intestinal environment less supportive of epithelial maintenance and recovery. Reductions in indole-3-acetic acid, indole-3-aldehyde, tryptamine, and indole-3-propionic acid further indicate disruption of tryptophan-derived metabolic signaling. Several of these metabolites regulate epithelial integrity and mucosal immunity through receptors including the aryl hydrocarbon receptor and pregnane X receptor (Venkatesh et al., 2014; Scott et al., 2020). Melphalan also reduced primary and secondary bile acids, which function as signaling mediators through FXR and TGR5 and contribute to epithelial, inflammatory, and metabolic homeostasis (Wahlström et al., 2016; Fiorucci and Distrutti, 2015). Consistent with previous evidence of impaired ileal bile-acid handling during melphalan-induced mucosal injury (Wardill et al., 2021), reductions in taurocholate, cholic acid, deoxycholic acid, and lithocholic acid support broad disruption of intestinal bile-acid homeostasis.

The interventions produced distinct patterns of metabolic recovery. FOS increased several SCFAs, consistent with its function as a fermentable prebiotic substrate and previous evidence of improved barrier function and SCFA availability in chemotherapy-induced mucositis models (Carvalho et al., 2021). The commercial probiotic preparation increased selected SCFAs, bile acids, and indole-derived metabolites, accompanied by improvements in circulating barrier-associated biomarkers. Reported antioxidant, anti-inflammatory, and epithelial barrier-supporting properties of *L. paracasei* Shirota (Finamore et al., 2018) provide a rationale for investigating preparations containing this strain, although the observed effects cannot be attributed exclusively to the strain. In contrast, chlorophyllin produced a more selective metabolic response, significantly increasing taurocholate, indole-3-propionic acid, and indole-3-acetic acid relative to melphalan alone, while no statistically significant differences were observed in the six measured SCFAs. Its effects on physiological and barrier-associated outcomes suggest possible contributions beyond changes in the measured metabolites. Previous reports of oxidative and autophagy-associated pathway modulation (Zhang et al., 2022) provide potential mechanisms for future investigation, but these mechanisms were not established in the present study.

Intervention-associated changes in cecal metabolites occurred alongside improvements in intestinal barrier-associated outcomes. At the group level, changes in acetate, butyrate, and indole-3-acetic acid accompanied higher plasma citrulline and lower circulating LPS, although intervention-associated differences in butyrate were not statistically significant. These descriptive patterns do not establish a causal relationship between individual metabolites and barrier recovery. At the individual-animal level, plasma citrulline was inversely associated with circulating LPS; this pooled association may partly reflect differences between treatment groups. Physiological measurements suggested partial attenuation of melphalan-associated body-weight loss and small-intestinal shortening. Descriptive Western blot findings suggested partial reversal of melphalan-associated changes in claudin-1, TLR2, and SOD1 expression following chlorophyllin treatment, whereas phosphorylated NF-κB showed modest variation across groups. Together, these findings indicate concurrent changes in physiological, circulating, molecular, and metabolic measures following intervention treatment. Histopathological abnormalities were observed in the melphalan-only group compared with largely normal vehicle-control findings. These abnormalities persisted in the intervention groups at Day +3, without consistent histological improvement despite improvements in physiological and plasma-marker outcomes. Later time points would be needed to determine the subsequent course of these structural changes.

Targeted metabolomic analysis included three biological samples per group because cecal samples containing <50 mg of material were excluded before analysis. This small sample size limits statistical power and the precision of effect estimates; therefore, the observed metabolic patterns should be considered exploratory and require confirmation in larger studies. Although targeted metabolite profiling provided information on the intestinal metabolic environment, microbial composition and diversity were not directly assessed. The observed responses therefore reflect changes in measured cecal metabolite profiles, while their relationship to microbiome changes remains to be investigated. Because the probiotic intervention was administered as a commercial fermented-milk preparation containing *L. paracasei* Shirota, the respective contributions of the bacterial strain and the fermented-milk matrix to the observed effects could not be distinguished.

The differing patterns of metabolite changes observed with FOS, the commercial probiotic preparation, and chlorophyllin suggest that their effects may involve different biological mechanisms. However, the present study does not establish these mechanisms or a causal relationship between metabolite changes and intestinal protection. Overall, the findings support further evaluation of these interventions for mitigating melphalan-associated intestinal toxicity, with the exploratory metabolite findings requiring confirmation in larger studies.

## Data Availability

The original contributions presented in the study are included in the article/Supplementary material, further inquiries can be directed to the corresponding author.

## Supplementary material

Supplementary Data Sheet S1 LC–MS/MS analytical parameters for targeted cecal metabolite profiling, including retention times, precursor/product-ion transitions, cone voltages, and collision energies.

Supplementary Data Sheet S2 Method-development, calibration, and analytical-performance data for the targeted LC–MS/MS metabolite assays.

Supplementary Data Sheet S3 Details of metabolite reference standards, isotopically labeled internal standards, and derivatization procedures used for targeted LC–MS/MS analysis.

Supplementary Data Sheet S4 Individual-animal small-intestinal histopathological findings and scoring following melphalan administration and intervention treatments.
