# Supplementary material for: Prebiotic, probiotic, and chlorophyllin interventions differentially attenuate melphalan-induced intestinal injury and modulate gut metabolite profiles
Source: Front Microbiol. 2026 Oct 29;17:2003316. doi: 10.3389/fmicb.2026.2003316 (PMC13755934; doi:10.3389/fmicb.2026.2003316)
Supplement: Supplementary file 1 [file Data_Sheet_1.PDF]

# LC-MS/MS Protocol for Fecal Metabolite Analysis

LC-MS/MS Protocol for Targeted Quantification of SCFAs, Bile Acids, and Indole Derivatives in Fecal Samples

**Reagents and Standards**

Methanol (LC-MS grade)
Acetonitrile (LC-MS grade)
Formic acid (LCMS grade)
Ammonium Formate (LCMS grade)

**Instrument Model :**

Xevo™ TQ Absolute triple quadrupole mass spectrometer (Waters Corporation, Milford, MA, USA) equipped with an electrospray ionization (ESI) source and operated in MRM mode. Data acquisition and processing were performed using MassLynx™ software.

**Calibration and linearity and quantification**

Calibration curves were generated using matrix-matched standards and fitted by weighted ($1/x^2$) least-squares linear regression, demonstrating linear responses ($r^2 \geq 0.99$) across the validated concentration ranges. For bile acids, deoxycholic acid showed linearity from 0.5 to 1600 µg/g dry weight, while lithocholic acid, taurocholate, and cholic acid were linear over 1.0 to 267 µg/g dry weight. Indole acid derivatives, including indole-3-propionic acid, indoxyl sulfate, tryptamine, indole-3-aldehyde, and indole-3-acetic acid, exhibited linearity across 0.5 to 267 µg/g dry weight. Short-chain fatty acids (acetate, propionate, butyrate, iso-butyrate, valerate, iso-valerate, and hexanoate) were linear over 3 to 80,000 µg/g dry weight. Isotopically labelled internal standards (0.10 µg/g dry weight) were added to all calibration standards, quality control samples, and study samples to enable isotope-dilution quantification.

**Bile acids**

Deoxycholic acid (DCA): 0.5–1600 µg/g DW
Lithocholic acid, taurocholate, and cholic acid: 1.0–267 µg/g DW

**Indole acid derivatives**

Indole-3-propionic acid, indoxyl sulfate, tryptamine, indole-3-aldehyde, and indole-3-acetic acid: 0.5–267 µg/g DW

**Short-chain fatty acids (SCFAs)**

# LC-MS/MS Protocol for Fecal Metabolite Analysis

Acetate, propionate, butyrate, iso-butyrate, valerate, iso-valerate, and hexanoate:
3–80,000 µg/g DW

**Derivatization Stock solutions preparations (store at −20 °C)**
3-NPH: 200 mM in acetonitrile : water (1:1)v/v (prepare fresh or store short term)
EDC·HCl: 200 mM in water (prepare fresh immediately before use; EDC decomposes)

**Sample Preparation**

1. About 20 mg of fecal sample into a 2 mL tube.
2. 400 µL of Acetonitrile : water (1:1, v/v) was added
3. Vortexed for 2 minutes and was sonicated for 15 minutes
4. The sample was the centrifuged at 10000rpm for 10 minutes
5. Collect supernatant
    a. For Bile acids & Indole derivatives : 10 µL of the supernatant was taken and further diluted to 200µL with diluent and injected to the system.
    b. For SCFA's derivatization.
        i. 100µL of the supernatant was mixed with 150 µL 3-NPH (200mM) and 150 µL EDC (120 mM).
        ii. Incubated at 40°C for 30 minutes.
        iii. Quench with 600µL of diluent
        iv. Then centrifuged and collected supernatant and injected into the system.

**Sample preparation and metabolite extraction**
Approximately 20 mg of fecal sample (dry weight) was accurately weighed into a 2 mL microcentrifuge tube. Metabolites were extracted by adding 400 µL of acetonitrile:water (1:1, v/v), followed by vortex mixing for 2 min and sonication for 30 min. Samples were centrifuged at 10,000 rpm for 10 min, and the resulting supernatant was collected.

**Analysis of bile acids and indole derivatives**
For bile acid and indole derivative analysis, 10 µL of the supernatant was transferred to a fresh tube and spiked with the isotopically labelled internal standards cholic acid-d4 and indole-d7, respectively. The extract was diluted to a final volume of 200 µL with acetonitrile:water (1:1, v/v) (20-fold dilution) prior to LC–MS/MS analysis.

**SCFA derivatization and analysis**
For short-chain fatty acid (SCFA) analysis, 100 µL of the supernatant was spiked with acetic acid-d4 and derivatized using 3-nitrophenylhydrazine (3-NPH, 200 mM) and EDC (120 mM). The derivatization reaction was carried out at 40 °C for 30 min and quenched

# LC-MS/MS Protocol for Fecal Metabolite Analysis

with 600 µL of acetonitrile:water (1:1, v/v). Following centrifugation, the supernatant was analyzed by LC–MS/MS.

**Chromatography Conditions :**

Column : Acquity UPLC HSS T3 (100 × 2.1 mm, 1.8 µm)
Flow rate : 0.3 mL/min
Column temperature : 40°C
Injection volume : 5 µL
Diluent : Acetonitrile : water (1:1, v/v)
Mobile Phases : A: 10mM Ammonium Formate + 0.1% formic acid
Mobile Phase B : Methanol
Gradient (Time/%B) : 0/5, 2/5, 3/50, 4/50, 6/75, 8/75, 9/90, 11/90, 11.1/5, 15/5

**Mass Spectrometry Conditions**

Ionization Source : ESI
Polarity : Positive and Negative mode
Capillary voltage : 3.2 kV
Cone voltage : 25V
Source Temperature : 150°C
Desolvation temperature : 450°C
Desolvation gas flow : 900 L/hr
Cone gas flow : 50 L/hr

| Compound | RT (min) | Neutral mass (Da) | Parent (Q1) m/z | Daughter (Q3) m/z | Cone (V) | Collison (eV) |
|---|---|---|---|---|---|---|
| **Bile Acid Derivatives** | | | | | | |
| Deoxycholic acid | 10.22 | 392.10 | 391.30 $[M-H]^-$ | 391.30 | 60 | 5 |
| Lithocholic acid | 10.85 | 376.10 | 375.10 $[M-H]^-$ | 375.10 | 25 | 15 |
| Taurocholate | 7.16 | 514.10 | 514.30 $[M-H]^-$ | 514.30 | 60 | 5 |
| Cholic acid | 9.26 | 407.10 | 407.30 $[M-H]^-$ | 407.30 | 60 | 5 |
| Cholic acid d4 | 9.23 | 411.10 | 411.20 $[M-H]^-$ | 411.20 | 60 | 5 |
| **Indole Acid Derivatives** | | | | | | |
| Indole-3-propionic acid | 5.35 | 189.10 | 190.1 $[M+H]^+$ | 130.10 | 25 | 18 |

## LC-MS/MS Protocol for Fecal Metabolite Analysis

| | | | | | | |
|---|---|---|---|---|---|---|
| Indoxyl sulfate | 3.76 | 213.10 | 212.0 $[M-H]^-$ | 212.10 | 25 | 5 |
| Tryptamine | 3.89 | 160.10 | 161.10 $[M+H]^+$ | 144.10 | 20 | 12 |
| Indole-3-aldehyde | 4.62 | 145.10 | 146.10 $[M+H]^+$ | 118.10 | 25 | 12 |
| Indole-3-acetic acid | 4.75 | 175.10 | 176.07 $[M+H]^+$ | 130.10 | 25 | 14 |
| Indole d7 | 5.45 | 124.10 | 125$[M+H]^+$ | 97.10 | 25 | 22 |
| **Short Chain Fatty Acids derivatize with 3-NPH and EDC** | | | | | | |
| Acetate | 4.15 | 195.06 | 194.10 $[M-H]^-$ | 137.01 | 25 | 18 |
| Butyrate | 4.96 | 223.09 | 222.10 $[M-H]^-$ | 137.10 | 25 | 18 |
| Isobutyrate | 4.96 | 223.09 | 222.10 $[M-H]^-$ | 137.10 | 25 | 18 |
| Propionate | 4.50 | 209.08 | 208.10 $[M-H]^-$ | 137.10 | 25 | 18 |
| Hexonate | 6.41 | 251.12 | 250.10 $[M-H]^-$ | 136.93 | 25 | 18 |
| Isovalerate | 5.82 | 237.11 | 236.10 $[M-H]^-$ | 137.01 | 25 | 18 |
| Valerate | 5.80 | 237.11 | 236.10 $[M-H]^-$ | 136.87 | 25 | 18 |
| Acetic acid d4 | 4.17 | 199.10 | 198.10 $[M-H]^-$ | 137.10 | 25 | 18 |

# LC-MS/MS Protocol for Fecal Metabolite Analysis

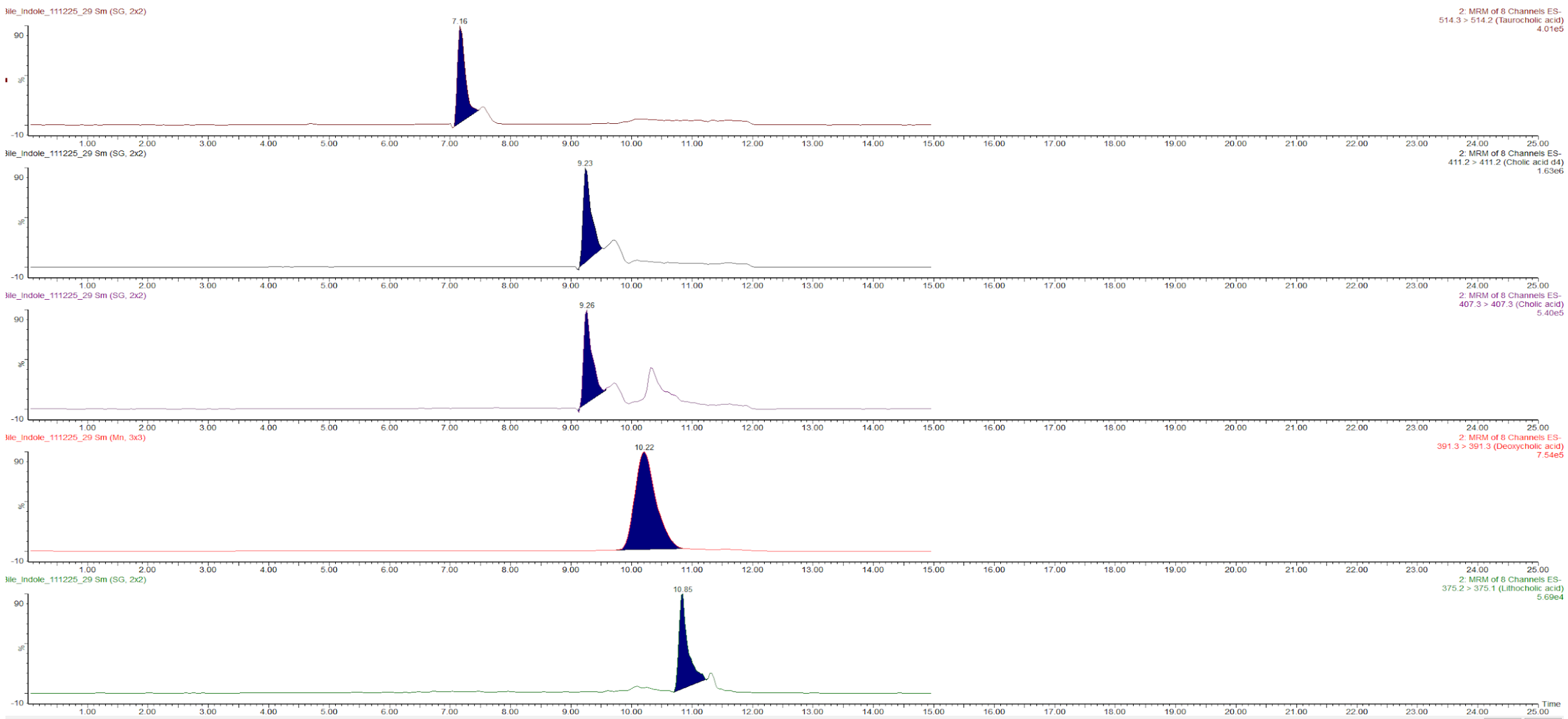


Fig 1 : Bile Acid Derivatives MRM

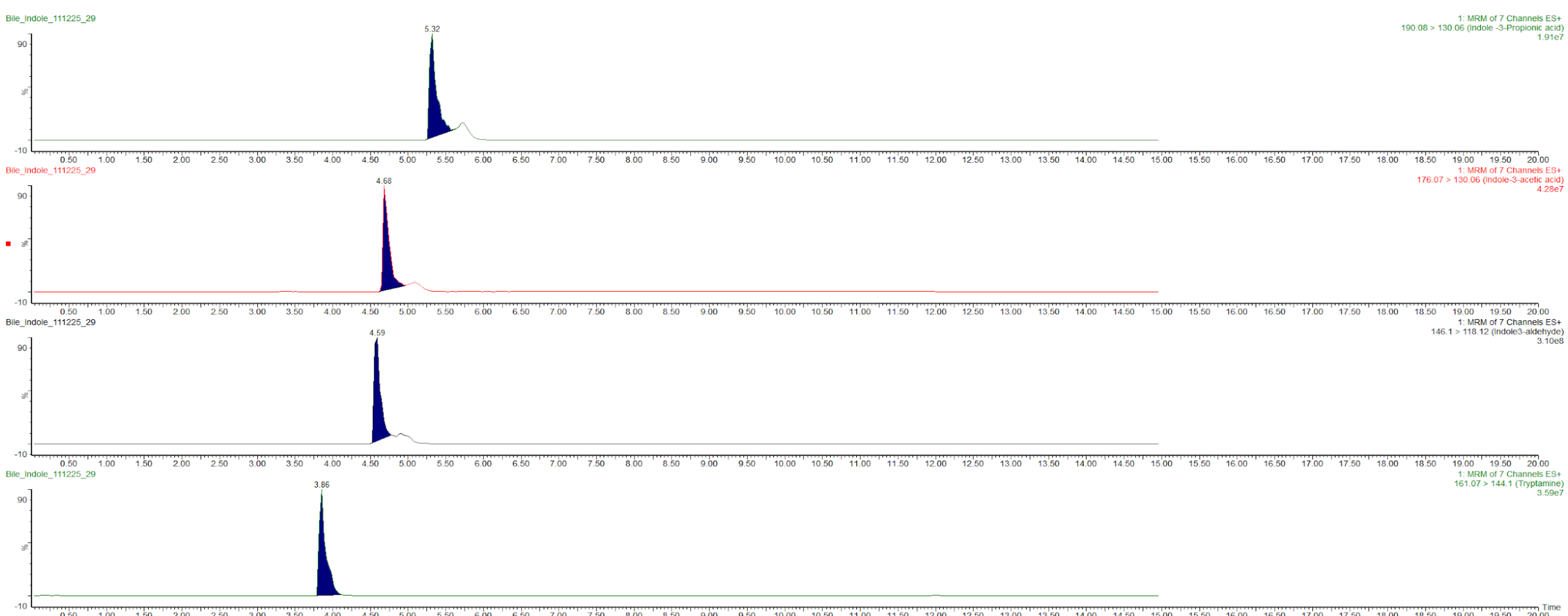


Fig 2 : Indole acid Derivatives MRM

# LC-MS/MS Protocol for Fecal Metabolite Analysis

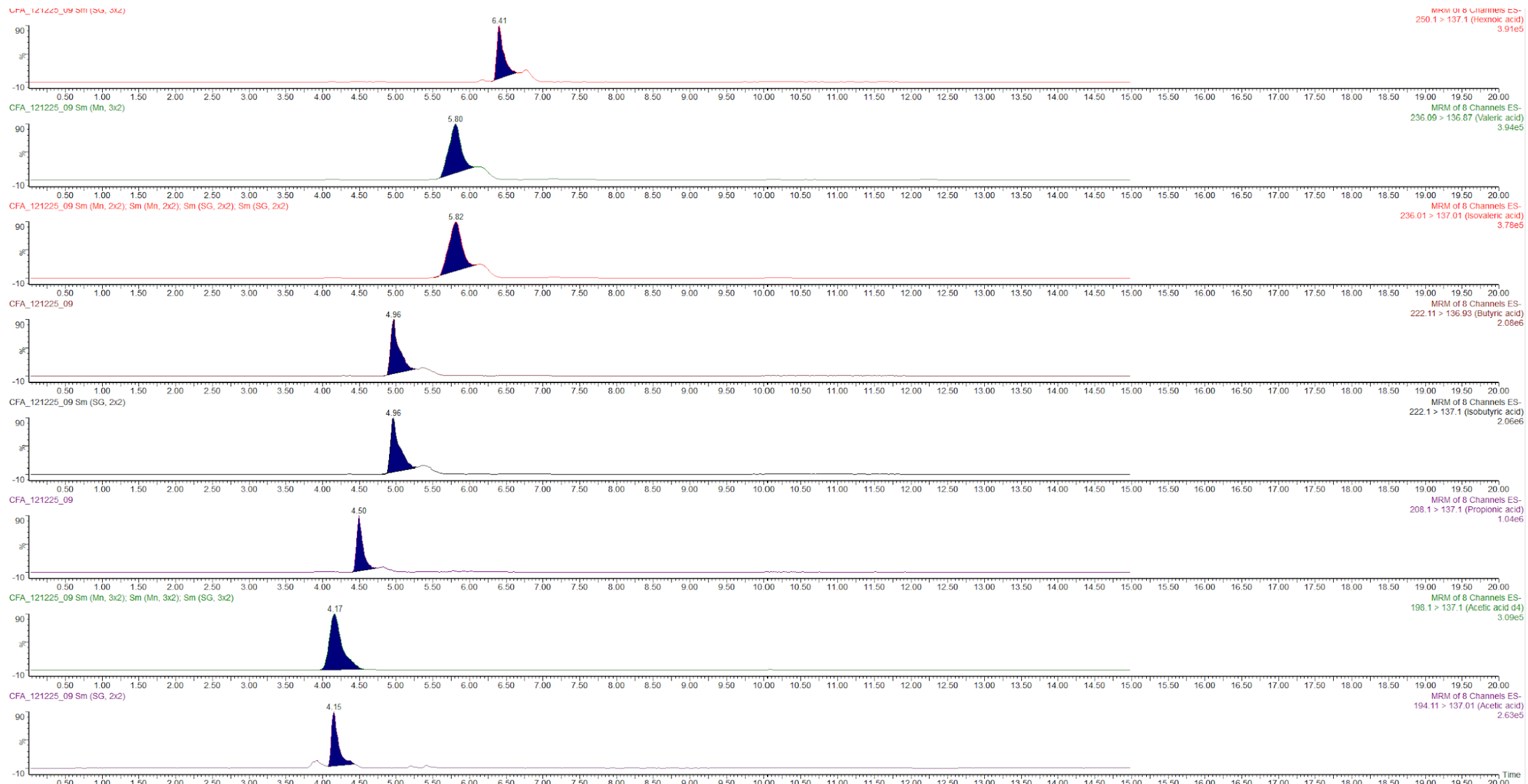


Fig 3 : SCFA Derivatives MRM
