# Supplementary material for: Prebiotic, probiotic, and chlorophyllin interventions differentially attenuate melphalan-induced intestinal injury and modulate gut metabolite profiles
Source: Front Microbiol. 2026 Oct 29;17:2003316. doi: 10.3389/fmicb.2026.2003316 (PMC13755934; doi:10.3389/fmicb.2026.2003316)
Supplement: Supplementary file 2 [file Data_Sheet_2.PDF]

LCMS Gut Metabolites Protocol

LC-MS/MS Protocol for Targeted Quantification of Bile Acids and Indole Derivatives

Reagents and Standards

Acetonitrile (LC-MS grade)
Formic acid (LCMS grade)
Internal Standards:
- Cholic acid-d4 (for bile acids)
- Indole-d7 (for indole derivatives)

Diluent : ACN : Water (50:50)v/v

Standard Solution Preparations : 100ng/mL each in diluent

Chromatography Conditions :

Column : Acquity UPLC HSS T3 (100 × 2.1 mm, 1.8 µm)
Flow rate : 0.4 mL/min
Column temperature : 40°C
Injection volume : 5 µL
Runtime : 15 mins
Mobile Phases A : 10mM Ammonium Formate + 0.1% formic acid
Mobile Phase B : Acetonitrile

Gradient:

Time (min)	Mobile Phase A%	Mobile Phase B%
0	95	5
2	95	5
5	5	70
6	10	90
10	10	90
10.1	95	5
15	95	5

LCMS Gut Metabolites Protocol

Mass Spectrometry Conditions

Ionization Source	: ESI
Curtain Gas	: 35 psi
Collision Gas	: Medium
IonSpray Voltage	: +4500 V (positive) / -4500 V (negative)
Temperature	: 500°C
Ion Source Gas 1	: 50 psi
Ion Source Gas 2	: 50 psi
Declustering Potential	: -100 v/ 100v
Entrance Potential	: -10v/ 10v
Collision Cell Exit Potential	: -10v/ 10v

MRM Transitions

Compound	Mode	Q1 (m/z)	Q3 (m/z)	CE	Structure
**Bile Acid Derivatives**					
Deoxycholic acid	Negative	391.3	345.2	-30	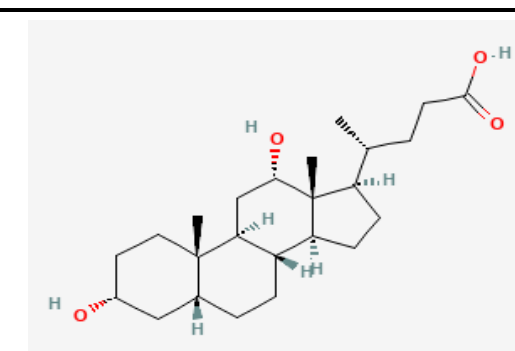
Lithocholic acid	Negative	375.2	357.1	-30	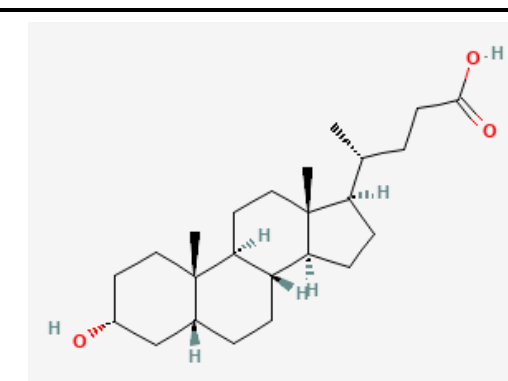
Taurocholate /Taurocholic Acid	Negative	514.3	411.5/228.18	-40	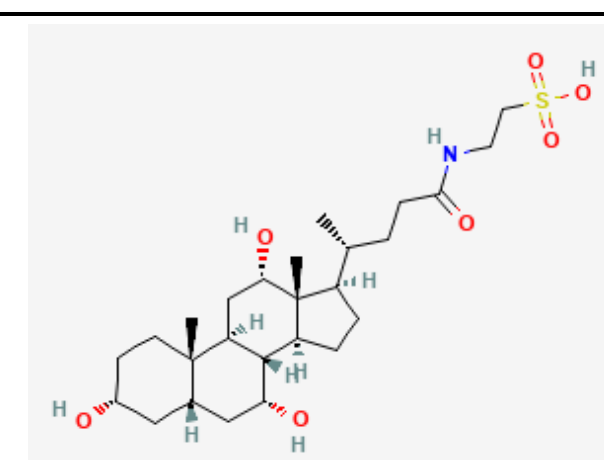
Cholic acid	Negative	407.3	343.2/289.2	-45	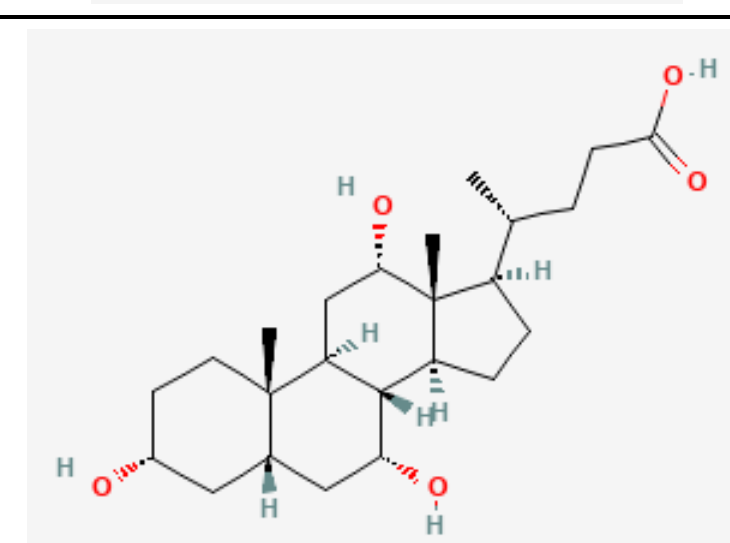

LCMS Gut Metabolites Protocol

**Indole Acid Derivatives**					
Indole-3-propionic acid	Positive	190.1	130.1	20	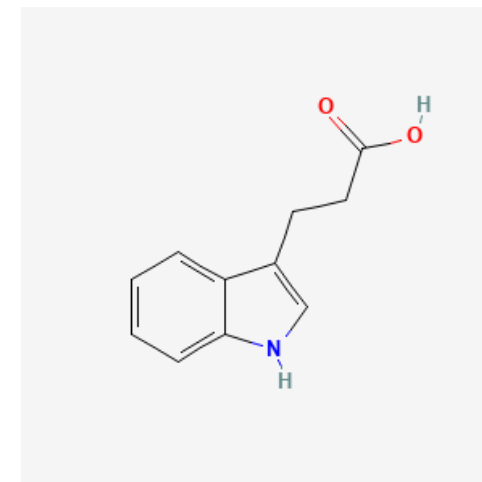
Indoxyl sulfate	Negative	212.0	132.0	-45	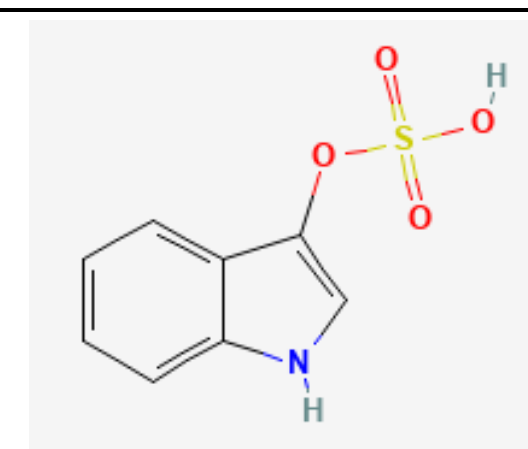
Tryptamine	Positive	161.1	144.1	15	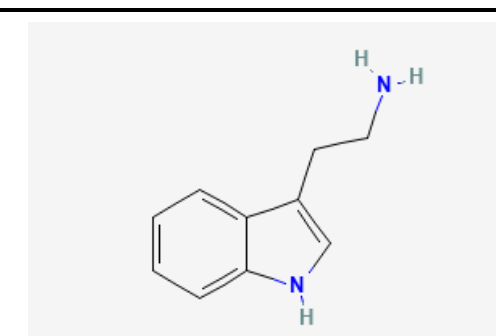
Indole-3-aldehyde	Positive	146.1	118.1	20	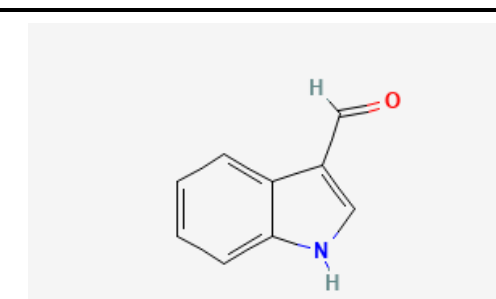
Indole-3-acetic acid	Negative	174.1	142	-15	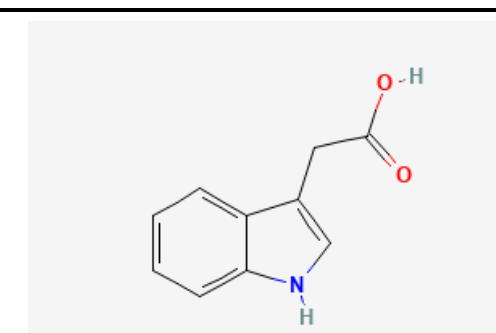
Internal Standard					
Cholic acid d4	Neg	411.2	391	-25	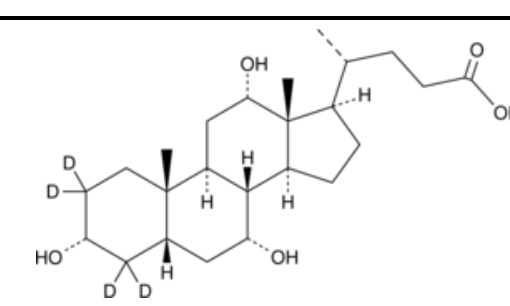
Indole d7 - 128	Pos	124	NA	NA	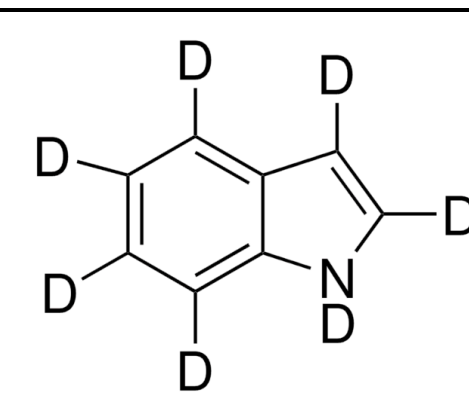
