# Supplementary material for: Prebiotic, probiotic, and chlorophyllin interventions differentially attenuate melphalan-induced intestinal injury and modulate gut metabolite profiles
Source: Front Microbiol. 2026 Oct 29;17:2003316. doi: 10.3389/fmicb.2026.2003316 (PMC13755934; doi:10.3389/fmicb.2026.2003316)
Supplement: Supplementary file 3 [file Data_Sheet_3.pdf]

Category	Standard/reagent	Manufacturer	Supplied batch/lot number or identifier
Bile acids	Deoxycholic acid	Sigma-Aldrich	BCCH8920
Bile acids	Lithocholic acid	Sigma-Aldrich	BCCL8897
Bile acids	Taurocholic acid sodium salt hydrate	Sigma-Aldrich	0000305445
Bile acids	Cholic acid	Sigma-Aldrich	0000587142
Internal standard	Cholic acid-d_4	Toronto Research Chemicals	TRC-C432603
Tryptophan-derived metabolites	Indole-3-propionic acid	Sigma-Aldrich	BCCJ3021
Tryptophan-derived metabolites	Indoxyl sulfate potassium salt	Sigma-Aldrich	BCCL0546
Tryptophan-derived metabolites	Tryptamine	Sigma-Aldrich	BCCK5363
Tryptophan-derived metabolites	Indole-3-carboxaldehyde	Sigma-Aldrich	STBL2801
Tryptophan-derived metabolites	Indole-3-acetic acid	Sigma-Aldrich	BCCG2092
Internal standard	Indole-d_7	Toronto Research Chemicals	TRC-I577331
SCFAs	Acetic acid	Sigma-Aldrich	BCCK7393
SCFAs	Butyric acid	Sigma-Aldrich	SHBQ6137
SCFAs	Isobutyric acid	Sigma-Aldrich	MKCT9170
SCFAs	Propionic acid	Sigma-Aldrich	BCCL2135
SCFAs	Hexanoic acid	Sigma-Aldrich	MKCS2390
SCFAs	Isovaleric acid	Sigma-Aldrich	STBL1676
SCFAs	Valeric acid	Sigma-Aldrich	SHBT0827
Internal standard	Acetic acid-d_4	Toronto Research Chemicals	TRC-A167657
Derivatization reagent	3-Nitrophenylhydrazine hydrochloride	Sigma-Aldrich	STBL4099
Derivatization reagent	1-(3-Dimethylaminopropyl)-3-ethylcarbodiimide hydrochloride (EDC)	BLD Pharm	GMP480
