# Supplementary material for: Prebiotic, probiotic, and chlorophyllin interventions differentially attenuate melphalan-induced intestinal injury and modulate gut metabolite profiles
Source: Front Microbiol. 2026 Oct 29;17:2003316. doi: 10.3389/fmicb.2026.2003316 (PMC13755934; doi:10.3389/fmicb.2026.2003316)
Supplement: Supplementary file 4 [file Data_Sheet_4.PDF]

Treatment group	Animal	Surface lesions/villous blunting	Crypt regeneration	Crypt epithelial cell apoptosis	Crypt loss	Lamina propria inflammation	Mucosal ulceration	Villous shortening
Vehicle control	F1	0	0	0	0	0	0	0
Vehicle control	F2	Superficial lesion	0	0	0	0	0	0
Vehicle control	F3	0	0	0	0	0	0	0
Vehicle control	F4	0	0	0	0	0	0	0
Vehicle control	F5	Autolysed	0	0	0	0	0	0
Vehicle control	F6	0	0	0	0	0	0	0
Melphalan only	F7	0	0	0	0	1	1	0
Melphalan only	F8	0	0	0	0	0	0	0
Melphalan only	F9	0	0	0	0	1	1	1
Melphalan only	F10	1	0	0	0	1	1	0
Melphalan only	F11	0	0	0	0	0	0	0
Melphalan only	F12	0	0	0	0	0	0	0
Melphalan + FOS	F13	0	1 (mitosis)	0	1	2	2	2
Melphalan + FOS	F14	0	0	0	1	1	1	0
Melphalan + FOS	F15	0	0	0	1	0	2	0
Melphalan + FOS	F16	0	0	0	1	1	1	0
Melphalan + FOS	F17	0	0	0	2	1	2	1
Melphalan + FOS	F18	0	0	0	1	1	1	0
Melphalan + probiotic	F19	0	0	0	0	1	1	1
Melphalan + probiotic	F20	NA	NA	NA	NA	NA	NA	NA
Melphalan + probiotic	F21	0	0	0	1	0	1	0
Melphalan + probiotic	F22	0	0	0	1	2	2	2
Melphalan + probiotic	F23	0	0	0	0	2	2	2
Melphalan + probiotic	F24	0	0	0	0	0	0	0
Melphalan + chlorophyllin	F25	0	0	0	1	0	1	0
Melphalan + chlorophyllin	F26	0	0	0	0	0	0	0
Melphalan + chlorophyllin	F27	0	0	0	1	1	1	0
Melphalan + chlorophyllin	F28	0	0	0	1	2	2	0
Melphalan + chlorophyllin	F29	0	0	0	1	2	1	0
Melphalan + chlorophyllin	F30	0	0	0	2	1	2	0

Supplementary Data Table S4. Histopathological assessment of H&E-stained small-intestinal sections from the main intervention experiment. Individual-animal findings are presented for vehicle control, melphalan only, and melphalan plus FOS, probiotic preparation, or chlorophyllin groups. Features assessed included surface epithelial lesions/villous blunting, crypt regeneration, crypt epithelial cell apoptosis, crypt loss, lamina propria inflammation, mucosal ulceration, and villous shortening. A score of 0 indicates no abnormality detected. Scores 1 and 2 and qualitative observations are reproduced as recorded. NA indicates unavailable assessment following death during sample collection. “Autolysed” denotes tissue autolysis, which may limit interpretation. Findings are presented descriptively. H&E, hematoxylin and eosin; FOS, fructooligosaccharides. Histopathological scores were defined as follows: 0, no abnormality detected; 1, mild abnormality; and 2, moderate abnormality. NA indicates unavailable assessment.
