# Supplementary material for: Prebiotic, probiotic, and chlorophyllin interventions differentially attenuate melphalan-induced intestinal injury and modulate gut metabolite profiles
Source: Front Microbiol. 2026 Oct 29;17:2003316. doi: 10.3389/fmicb.2026.2003316 (PMC13755934; doi:10.3389/fmicb.2026.2003316)
Supplement: Supplementary file 5 [file Image_1.PDF]

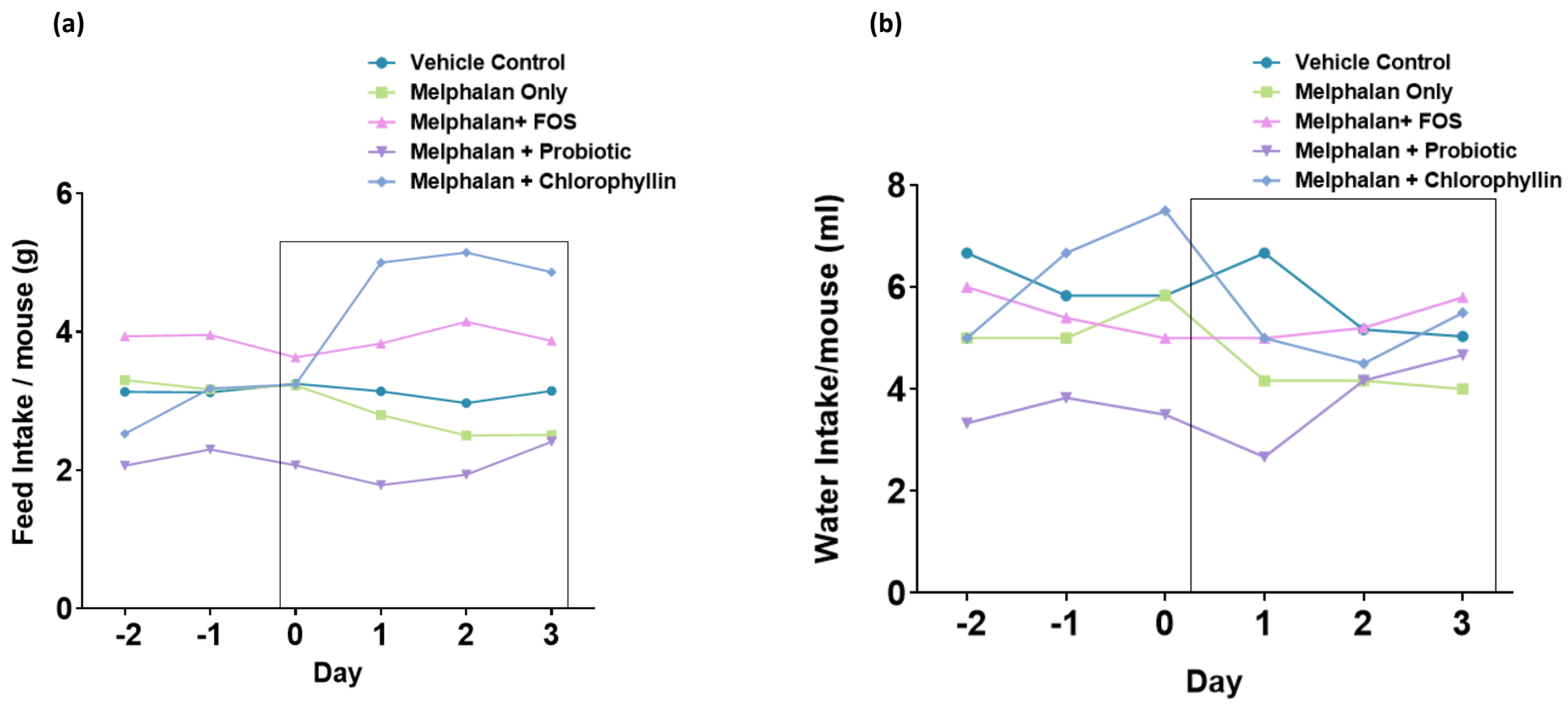

Supplementary Figure S1. Daily food and water intake before and after melphalan administration. (A) Food intake and (B) water intake, expressed per mouse per day. Intake was measured at the cage level and divided by the number of mice housed in each cage. Measurements were recorded from Day −2 to Day +3 in the vehicle control, melphalan-only, and melphalan plus FOS, probiotic, or chlorophyllin groups. The vertical line indicates melphalan administration on Day 0.

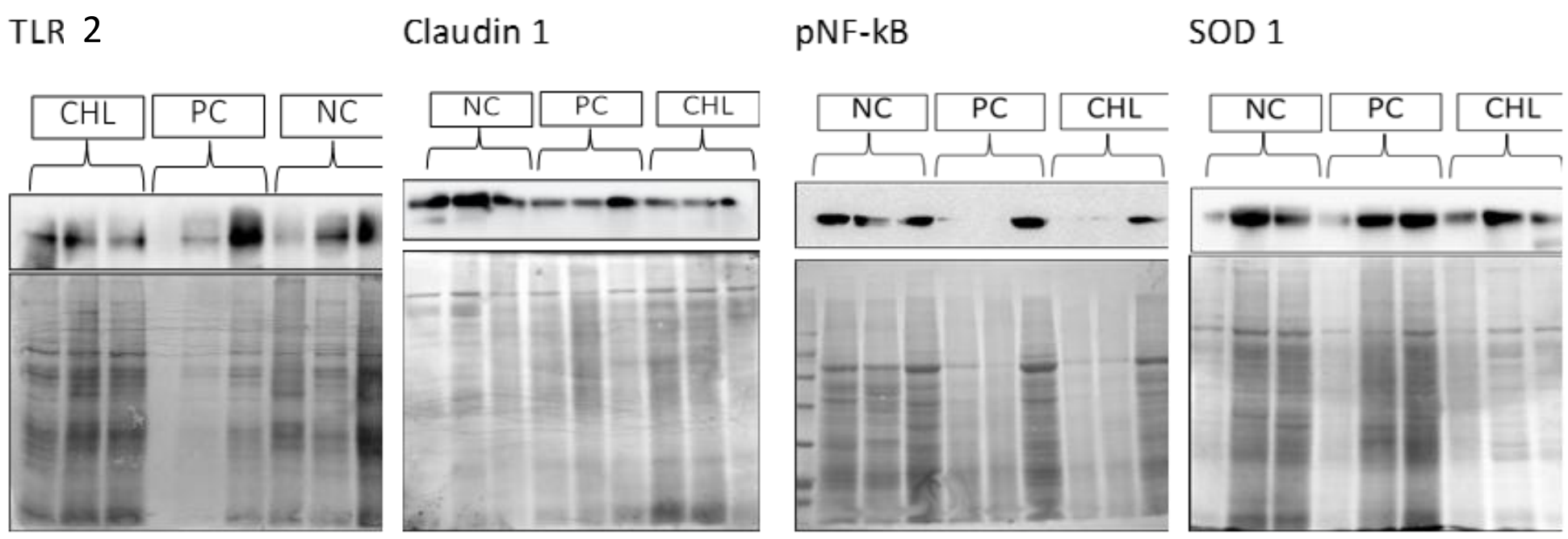

Supplementary Figure S 2. Representative Western blot images showing barrier- and inflammation-associated protein expression in small-intestinal tissue following melphalan administration and microbiome-directed interventions.
